# Gene Expression Classification of Colon Cancer into Molecular Subtypes: Characterization, Validation, and Prognostic Value

**DOI:** 10.1371/journal.pmed.1001453

**Published:** 2013-05-21

**Authors:** Laetitia Marisa, Aurélien de Reyniès, Alex Duval, Janick Selves, Marie Pierre Gaub, Laure Vescovo, Marie-Christine Etienne-Grimaldi, Renaud Schiappa, Dominique Guenot, Mira Ayadi, Sylvain Kirzin, Maurice Chazal, Jean-François Fléjou, Daniel Benchimol, Anne Berger, Arnaud Lagarde, Erwan Pencreach, Françoise Piard, Dominique Elias, Yann Parc, Sylviane Olschwang, Gérard Milano, Pierre Laurent-Puig, Valérie Boige

**Affiliations:** 1 “Cartes d'Identité des Tumeurs” Program, Ligue Nationale Contre le Cancer, Paris, France; 2 Unité Mixte de Recherche S938, Centre de Recherche Hôpital Saint-Antoine, INSERM, Paris, France; 3 Université Pierre et Marie Curie–Paris 6, Paris, France; 4 Unité Mixte de Recherche 1037, Centre de Recherche en Cancérologie de Toulouse, Université de Toulouse III, INSERM, Toulouse, France; 5 Unité de Recherche Physiopathologie et Médecine Translationnelle EA 4438, Université de Strasbourg, Strasbourg, France; 6 Laboratoire de Biochimie et Biologie Moléculaire, Hôpital de Hautepierre, Hôpitaux Universitaires de Strasbourg, Strasbourg, France; 7 Laboratoire d'Oncopharmacologie EA 3836, Centre Antoine Lacassagne, Nice, France; 8 Clinique Saint-George, Nice, France; 9 Service d'Anatomie et Cytologie Pathologiques, Hôpital Saint-Antoine, Assistance Publique-Hôpitaux de Paris, Paris, France; 10 Centre Hospitalier Universitaire de Nice, Nice, France; 11 Hôpital Européen Georges Pompidou, Paris, France; 12 Unité Mixte de Recherche S910, Faculté de Médecine La Timone, INSERM, Marseille, France; 13 Centre de Ressources Biologiques, Hôpitaux Universitaires de Strasbourg, Strasbourg, France; 14 Service de Pathologie, Centre Hospitalier Universitaire, Dijon, France; 15 Institut Gustave Roussy, Villejuif, France; 16 Service de Chirurgie Générale et Digestive, Hôpital Saint-Antoine, Assistance Publique-Hôpitaux de Paris, Paris, France; 17 Pôle DACCORD, Hôpital La Timone, Marseille, France; 18 Département d'Oncologie, Hôpital Clairval, Marseille, France; 19 Département de Gastroentérologie, Hôpital Ambroise Paré, Marseille, France; 20 Unité Mixte de Recherche S775, Paris Sorbonne Cité, Université Paris Descartes, INSERM, Paris, France; Fred Hutchinson Cancer Research Center, United States of America

## Abstract

**Background:**

Colon cancer (CC) pathological staging fails to accurately predict recurrence, and to date, no gene expression signature has proven reliable for prognosis stratification in clinical practice, perhaps because CC is a heterogeneous disease. The aim of this study was to establish a comprehensive molecular classification of CC based on mRNA expression profile analyses.

**Methods and Findings:**

Fresh-frozen primary tumor samples from a large multicenter cohort of 750 patients with stage I to IV CC who underwent surgery between 1987 and 2007 in seven centers were characterized for common DNA alterations, including *BRAF*, *KRAS*, and *TP53* mutations, CpG island methylator phenotype, mismatch repair status, and chromosomal instability status, and were screened with whole genome and transcriptome arrays. 566 samples fulfilled RNA quality requirements. Unsupervised consensus hierarchical clustering applied to gene expression data from a discovery subset of 443 CC samples identified six molecular subtypes. These subtypes were associated with distinct clinicopathological characteristics, molecular alterations, specific enrichments of supervised gene expression signatures (stem cell phenotype–like, normal-like, serrated CC phenotype–like), and deregulated signaling pathways. Based on their main biological characteristics, we distinguished a deficient mismatch repair subtype, a *KRAS* mutant subtype, a cancer stem cell subtype, and three chromosomal instability subtypes, including one associated with down-regulated immune pathways, one with up-regulation of the Wnt pathway, and one displaying a normal-like gene expression profile. The classification was validated in the remaining 123 samples plus an independent set of 1,058 CC samples, including eight public datasets. Furthermore, prognosis was analyzed in the subset of stage II–III CC samples. The subtypes C4 and C6, but not the subtypes C1, C2, C3, and C5, were independently associated with shorter relapse-free survival, even after adjusting for age, sex, stage, and the emerging prognostic classifier Oncotype DX Colon Cancer Assay recurrence score (hazard ratio 1.5, 95% CI 1.1–2.1, *p* = 0.0097). However, a limitation of this study is that information on tumor grade and number of nodes examined was not available.

**Conclusions:**

We describe the first, to our knowledge, robust transcriptome-based classification of CC that improves the current disease stratification based on clinicopathological variables and common DNA markers. The biological relevance of these subtypes is illustrated by significant differences in prognosis. This analysis provides possibilities for improving prognostic models and therapeutic strategies. In conclusion, we report a new classification of CC into six molecular subtypes that arise through distinct biological pathways.

*Please see later in the article for the Editors' Summary*

## Editors' Summary

**Background:**

Cancer of the large bowel (colorectal cancer) is the third most common cancer in men and the second most common cancer in women worldwide. Despite recent advances in the screening, diagnosis, and treatment of colorectal cancer, an estimated 608,000 people die every year from this form of cancer—8% of all cancer deaths. The prognosis and treatment options for colorectal cancer depend on five pathological stages (0–IV), each of which has a different treatment option and five year survival rate, so it is important that the stage is correctly identified. Unfortunately, pathological staging fails to accurately predict recurrence (relapse) in patients undergoing surgery for localized colorectal cancer, which is a concern, as 10%–20% of patients with stage II and 30%–40% of those with stage III colorectal cancer develop recurrence.

**Why Was This Study Done?:**

Previous studies have investigated whether there are any possible gene expression profiles (identified through microarray techniques) that can help predict prognosis of colorectal cancer, but so far, there have been no firm conclusions that can aid clinical practice. In this study, the researchers used genetic information from a French multicenter study to identify a standard, reproducible molecular classification based on gene expression analysis of colorectal cancer. The authors also assessed whether there were any associations between the identified molecular subtypes and clinical and pathological factors, common DNA alterations, and prognosis.

**What Did the Researchers Do and Find?:**

The researchers used genetic information from a cohort of 750 patients with stage I to IV colorectal cancer who underwent surgery between 1987 and 2007 in seven centers in France. The researchers identified relevant clinical and pathological staging information for each patient from the medical records and calculated recurrence-free survival (the time from surgery to the first recurrence) for patients with stage II or III disease. In the genetic analysis, 566 tumor samples were suitable—443 were used in a discovery set, to create the classification, and the remainder were used in a validation set, to test the classification. The researchers also used information from eight public datasets to validate their findings.

Using these methods, the researchers classified the colon cancer samples into six molecular subtypes (based on gene expression data) and, on further analysis and validation, were able to distinguish the main biological characteristics and deregulated pathways associated with each subtype. Importantly, the researchers found that that these six subtypes were associated with distinct clinical and pathological characteristics, molecular alterations, specific gene expression signatures, and deregulated signaling pathways. In the prognostic analysis based on recurrence-free survival, the researchers found that patients whose tumors were classified in one of two clusters (C4 and C6) had poorer recurrence-free survival than the other patients.

**What Do These Findings Mean?:**

These findings suggest that it is possible to classify colorectal cancer into six robust molecular subtypes that might help identify new prognostic subgroups and could provide a basis for developing robust prognostic genetic signatures for stage II and III colorectal cancer and for identifying specific markers for the different subtypes that might be targets for future drug development. However, as this study was retrospective and did not include some known predictors of colorectal cancer prognosis, such as tumor grade and number of nodes examined, the significance and robustness of the prognostic classification requires further confirmation with large prospective patient cohorts.

**Additional Information:**

Please access these websites via the online version of this summary at http://dx.doi.org/10.1371/journal.pmed.1001453.

- The American Cancer Society provides information about colorectal cancer and also about how colorectal cancer is staged
- The US National Cancer Institute also provides information on colon and rectal cancer and colon cancer stages

## Introduction

Despite advances in screening, diagnosis, and treatment, colorectal cancer (CRC) is the third most common cancer and the fourth-leading cause of cancer death worldwide [1]. Pathological staging is the only prognostic classification used in clinical practice to select patients for adjuvant chemotherapy [2]. However, pathological staging fails to predict recurrence accurately in many patients undergoing curative surgery for localized CRC. In fact, 10%–20% of patients with stage II CRC, and 30%–40% of those with stage III CRC, develop recurrence. Among the molecular markers that have been extensively investigated for colon cancer (CC) characterization and prognosis, microsatellite instability (MSI), caused by defective function of the DNA mismatch repair (MMR) system, is the only marker that was reproducibly found to be a significant prognostic factor in early CRC in both a meta-analysis and a prospective trial [3],[4]. Many studies have exploited microarray technology to investigate gene expression profiles (GEPs) in CRC in recent years, but no established signature has been found that is useful for clinical practice, especially for predicting prognosis [5]–[8]. GEP studies on CRC have been only poorly reproducible, possibly because CRC is composed of distinct molecular entities that may develop through multiple pathways on the basis of different molecular features [9]–[11]. As a consequence, there may be several prognostic signatures for CRC, each corresponding to a different entity. Indeed, GEP studies that include unsupervised hierarchical clustering, and integrated genetic/epigenetic analysis—including the more recent classification based on high-throughput methylome data [12]—have identified at least three distinct molecular subtypes of CC [7],[9]–[13]. Therefore, CC should no longer be considered as a homogeneous entity. However, the molecular classification of CC currently used, which is based on a few common DNA markers (MSI, CpG island methylator phenotype [CIMP], chromosomal instability [CIN], and *BRAF* and *KRAS* mutations) [9]–[11], needs to be refined, and a standard and reproducible molecular classification is still not available.

In this study, we exploited a large, multicenter, and extensively characterized series of CC samples to establish a robust molecular classification based on genome-wide mRNA expression analysis. Then we assessed the associations between molecular subtypes and clinicopathological factors, common DNA alterations, and prognosis. To confirm the robustness of the subtypes obtained, we further validated our molecular classification in a large independent set.

## Methods

### Ethics Committee Approval

The use of the tumor collection was approved by the following ethics committees and institutional boards: lle de France II (2008-135; AFSSAP 2008-A01058-47), Marseille (PHRC2005, COS-IPC of 27 September 2007), Strasbourg (Comité Consultatif de Protection des Personnes dans la Recherche Biomedicale d'Alsace, 2004-63 and CPP-EST4 [DC-2009-1016 and AC-2008-438]), the Human Research Ethics Committee of Saint-Antoine Hospital (INCa; TUM0203—project 2010-1-RT-02), the Toulouse Hospital board (CRB–Cancer Toulouse, DC-2008-463, AC-2008-820, CPP2), and Nice (PHRC1997, CHUNice-948). The informed consent of the patients was recorded as required by a French law in force until 2007. Since the last inclusion in this study was 2007, the standard hospital blanket consent was considered sufficient.

### Patients

The French national Cartes d'Identité des Tumeurs (CIT) program involves a multicenter cohort of 750 patients with stage I to IV CC who underwent surgery between 1987 and 2007 in seven centers. Fresh-frozen primary tumor tissue samples were retrospectively collected at the Institut Gustave Roussy (Villejuif), the Hôpital Saint Antoine (Paris), the Hôpital Européen Georges Pompidou (Paris), the Hôpital de Hautepierre (Strasbourg), the Hôpital Purpan (Toulouse), and the Institut Paoli-Calmettes (Marseille), and prospectively collected at the Centre Antoine Lacassagne (Nice). Patients who received preoperative chemotherapy and/or radiation therapy and those with primary rectal cancer were excluded from this study. Clinical and pathologic data were extracted from the medical records and centrally reviewed for the purpose of this study. Patients were staged according to the American Joint Committee on Cancer tumor node metastasis (TNM) staging system [2] and monitored for relapse (distant and/or locoregional recurrence; median follow-up of 51.5 mo). Patient and tumor characteristics are summarized in Table 1 and detailed in Table S1.

**Table 1:** Patient and tumor characteristics of the different sets.

Characteristics	CIT Cohort Patients (*n* = 750)	CIT Discovery Dataset (*n* = 443)	Validation Datasets		CIT Cohort *p*-Value	All Cohorts *p*-Value
			CIT (*n* = 123)	Public (*n* = 906)		
**Mean age (sd, range), years**	67 (14, 19–97)	67 (14, 22–97)	68 (12, 42–90)	68 (13, 23–95)	0.21	0.25
**Sex (male/female) (percent)**	429/321 (57/43)	237/206 (53/47)	73/50 (59/41)	347/330 (51/49)	0.24	0.24
**TNM stage (percent)**						
I	52 (7)	27 (6)	10 (8)	48 (11)	0.058	<0.001
II	351 (47)	198 (45)	66 (54)	205 (46)		
III	265 (35)	164 (37)	41 (33)	113 (25)		
IV	82 (11)	54 (12)	6 (5)	83 (18)		
NA	0	0	0	457		
**Location (percent)**						
Proximal	305 (41)	176 (40)	48 (39)	125 (51)	0.97	0.014
Distal	445 (59)	267 (60)	75 (61)	122 (49)		
NA	0	0	0	659		
**Adjuvant chemotherapy** a **(percent)**						
Yes	257 (42)^b^	161 (45)^b^	42 (40)^b^	91 (51)	0.42	0.31
No	357 (58)	200 (55)	64 (60)	87 (49)		
NA	2	1	6	140		
**Median follow-up (sd, range), months**	51.5 (37, 0–201)	50 (39, 0–201)	58 (37, 0–146)	48 (26, 0–143)	0.33	<0.001
**Relapse** a **(percent)**						
Yes	179 (29)	109 (30)	30 (29)	75 (24)	0.81	0.08
Distant/locoregional/both	149/23/7	83/22/4	29/0/1	—		
No	428 (71)	250 (70)	72 (71)	239 (76)		
NA	9	3	5	4		
**dMMR (percent)**	118/701 (17)	61/409 (15)	14/110 (13)	126/418 (30)	0.67	<0.001
**CIMP+ (percent)**	102/555 (18)	74/380 (19)	17/116 (15)	—	0.3	—
***KRAS*** **-mutant (percent)**	261/680 (38)	172/392 (41)	45/121 (37)	—	0.57	—
***BRAF*** **-mutant (percent)**	70/634 (11)	44/424 (11)	7/120 (6)	—	0.12	—
***TP53*** **-mutant (percent)**	226/451 (50)	135/245 (55)	55/106 (52)	—	0.66	—

*p*-Values are Chi-squared test *p*-values comparing the discovery and validation sets in the CIT cohort only and in all cohorts (excluding samples for which data were not available).

a Among patients with stage II–III CC.

b Only fluorouracil and folinic acid.

NA, not available; sd, standard deviation.

Of the 750 tumor samples of the CIT cohort, 566 fulfilled RNA quality requirements for GEP analysis (Figure S1). The 566 samples were split into a discovery set (*n* = 443) and a validation set (*n* = 123), well balanced for the main anatomoclinical characteristics (Table 1). The validation set also included 906 CC samples available from seven public datasets (GSE13067, GSE13294, GSE14333, GSE17536/17537, GSE18088, GSE26682, and GSE33113). These datasets corresponded to all available public datasets fulfilling the following criteria: available GEP data obtained using a similar chip platform (Affymetrix U133 Plus 2.0 chips) with raw data CEL files, and tumor location and either common DNA alteration (*n* = 457) and/or patient outcome (*n* = 449) data available. Within the discovery (*n* = 443) and the validation (*n* = 1,029) sets, 359 and 416 patients with stage II–III CC and documented relapse-free survival (RFS) were available for survival analysis, respectively (Figure S1). The dataset from The Cancer Genome Atlas (TCGA) [13], although obtained using a non-Affymetrix platform and therefore analyzed separately, was added to the validation set because of the extensive DNA alteration annotations provided for 152 CC samples.

### Gene Mutations, MMR Status, and CIMP Analysis

The seven most frequent mutations in codons 12 and 13 of *KRAS* were assessed as previously described [14]. The *BRAF* c.1799T>A (p.V600E) mutation was assessed by allelic discrimination using TaqMan probes and the same protocol as that for *KRAS* mutations. *TP53* mutations (exons 4–9) were assessed as previously described [15]. MSI was analyzed using a panel of five different microsatellite loci from the Bethesda reference panel [16]. MSI-high tumors were further classified as deficient MMR (dMMR), and both MSI-low and MSS tumors as proficient MMR (pMMR). CIMP status was determined using a panel of five markers (CACNA1G, IGF2, NEUROG1, RUNX3, and SOCS1) as previously described [17]. Experimental procedures are detailed in Text S1. Common DNA alterations are summarized in Table 1 and detailed in Table S1.

### Gene Expression Analysis

The GEP of 566 primary CC samples were determined on Affymetrix U133 Plus 2.0 chips. For 19 patients, adjacent non-tumor tissue (normal tissue [NT]) was also available and was tested. The methods used for RNA purification, quality control, fluorescent probe production, hybridization, and raw data processing were as previously described [18]. Each dataset was normalized independently in batches using the robust multi-array average method implemented in the R package affy [19]. For the CIT dataset, residual technical batch effects were corrected using the ComBat method implemented in the SVA R package [20]. Data are available via the NCBI Gene Expression Omnibus (http://www.ncbi.nlm.nih.gov/geo/; accession number GSE39582).

### Array-Based Comparative Genomic Hybridization Analysis

A total of 464 of the 750 primary CC samples from the CIT cohort could be analyzed for array-based comparative genomic hybridization (CGH) on a BAC array containing 4,434 bacterial artificial chromosome clones with a median resolution of 0.6 Mb. DNA labeling, hybridization, and data processing were as previously described [21]. CIN was defined from CGH profiles: samples with at least 20% gain or loss of whole chromosomes or fractions of chromosomes were scored as CIN+ (see Text S1 for details).

### Unsupervised Subtype Discovery Based on Gene Expression Analysis

Unsupervised classification of the discovery set was performed using hierarchical clustering (Ward linkage and 1 − Pearson correlation coefficient distance used) on the most variant class of probe sets (*n* = 1,459). To obtain a robust classification, we used a consensus unsupervised approach [22] implemented in the R package ConsensusClusterPlus. The consensus clusters were obtained from 1,000 resampling iterations of the hierarchical clustering, by randomly selecting a fraction of the samples and of the most variant probe sets (90%). The optimal number of clusters was selected according to the approach criteria detailed in Text S1.

### Validation Set Subtype Assignment

Validation datasets were independently assigned to GEP subtypes according to a standard distance-to-centroid approach [23]. A centroid-based predictor was built by a 10-fold cross-validation approach, resulting in the selection of the five top up-regulated and five top down-regulated genes specific to each subtype, yielding 57 genes (three genes were shared by two subtypes). The approach was implemented in the R package citccmst, and is detailed in Text S1.

### Molecular Subtype Characterization

The Chi-squared test and logistic regression were used to study associations between anatomoclinical features, common DNA alterations, and subtypes. Each molecular subtype was further characterized according to (i) GEP of NT counterparts from our dataset; (ii) previously published supervised signatures based on intestinal stem cell phenotype [24],[25], *BRAF* mutation [26], and serrated CRC phenotype [27], as described in Text S1; (iii) cancer-relevant signaling pathways retrieved from the Kyoto Encyclopedia of Genes and Genomes (see Text S1); and (iv) CGH alteration frequencies.

### Recurrence Risk Group Assignment according to Other Molecular Predictors

The ColoPrint and Oncotype DX prognostic classifiers [7],[8] were adapted and applied to our overall datasets as described in Text S1.

### Survival Analysis

Survival analysis was intentionally restricted to the subgroup of patients with stage II–III tumors because reliable prognostic biomarkers are most needed for these patients. Indeed, most stage I patients will not derive benefit from adjuvant chemotherapy because of their excellent prognosis after curative surgery, and most stage IV patients, already metastatic, will die from their disease and therefore should be analyzed independently for progression-free survival. RFS was defined as the time from surgery to the first recurrence and was censored at 5 y. Survival was analyzed according to the Kaplan-Meier method, and differences between survival distributions were assessed with the log-rank test. Univariate and multivariate models were computed using Cox proportional-hazards regression (R package survival) (see Text S1 for details).

## Results

### Unsupervised Analysis of Gene Expression Profiles Revealed Six Subtypes of Colon Cancer

**Figure 1:**
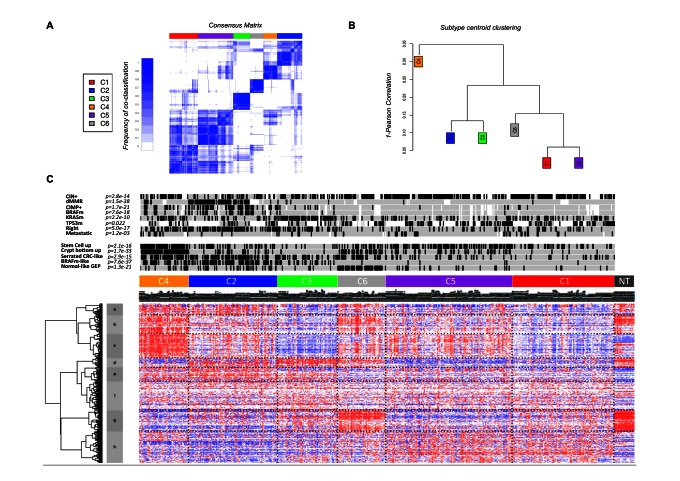
Unsupervised gene expression analysis of the discovery set of 443 colon cancers. (A) Consensus matrix heatmap defining six clusters of samples for which consensus values range from 0 (in white, samples never clustered together) to 1 (dark blue, samples always clustered together). (B) Distance between clusters according to the hierarchical clustering of the 1,459 probe sets based on the centroids of each cluster. (C) GEP heatmap of the 1,459 probe sets ordered by subtype, with annotations associated with each subtype.

Consensus unsupervised analysis of the GEP data from the 443 samples of the discovery set revealed six clusters of samples based on the most variant probe sets (*n* = 1,459): C1 (*n* = 95, 21%), C2 (*n* = 83, 19%), C3 (*n* = 56, 13%), C4 (*n* = 46, 10%), C5 (*n* = 118, 27%), and C6 (*n* = 45, 10%) (Figure 1; Table S2). The consensus matrix showed that C2, C3, C4, and C6 appeared as well-individualized clusters, whereas there was more classification overlap between C1 and C5 (Figure 1A). Based on cluster expression centroid classification and the gene expression heatmap (Figure 1B and 1C), cluster C4 appeared to be the most distinct. The other clusters subdivided into C2 and C3 on one side of the cluster expression centroid classification (Figure 1B), and C6, C5, and C1 on the other. The GEPs of C1 and C5 showed overlap but displayed slightly distinct gene deregulations. This was confirmed in the supervised selection of the cluster-discriminant probe sets shown in the gene expression heatmap in Figure S2 and detailed in Table S3.

### Clinical and Molecular Relevance of Colon Cancer Subtypes

Associations with anatomoclinical and DNA alterations data are shown in Figures 1C and S3A and in Table S4. Tumors classified as C1, C5, and C6 were more frequently CIN+, CIMP−, *TP53*-mutant, and distal (*p*<0.001), without any other molecular or clinicopathological features able to discriminate these three clusters clearly. Tumors classified as C2, C4, and C3 were more frequently CIMP+ (59%, 34%, and 18%, respectively, versus <5% in other clusters) and proximal. C2 was enriched for dMMR (68%) and *BRAF*-mutant tumors (40%). C3 was enriched for *KRAS*-mutant tumors (87%). No association between clusters and TNM stage was found, except enrichment for metastatic (31%) tumors in C4.

The analyses of CGH arrays revealed that CIN+ samples shared a typical DNA copy alteration pattern including +7, −8p, +8q, +13q, −17p, −18, +20q. Differences between subtypes mainly reflected their relative content of CIN+ samples. However, some specific alterations were observed for the two CIN subtypes, C5 (+2, +11, +17q) and C1 (−10q, −14q, −15q) (Figure S4).

### Signaling Pathways Associated with Colon Cancer Subtypes

**Figure 2:**
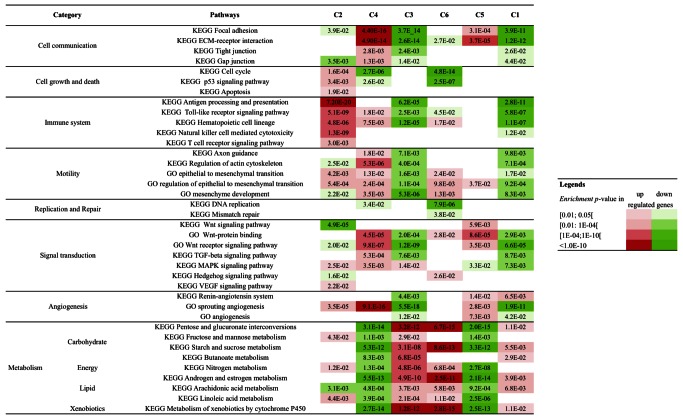
Signaling pathways associated with each molecular subtype. The enrichment of Kyoto Encyclopedia of Genes and Genomes (KEGG) and GeneOntology (GO) pathways and gene sets related to cancer hallmarks was tested in each subtype signature (1,000 top differentially up- and down-expressed genes, separately). The hypergeometric test *p*-values for enrichment in up- and down-regulated signatures are indicated in red and green, respectively. ECM, extracellular matrix.

We analyzed cancer-related signaling pathways from the Kyoto Encyclopedia of Genes and Genomes database for specific deregulation in each subtype signature (Figure 2). As expected, up-regulated immune system and cell growth pathways were found in C2, the subtype enriched for dMMR tumors. C4 and C6 both showed down-regulation of cell growth and death pathways and up-regulation of the epithelial–mesenchymal transition/motility pathways. Most signaling pathways were down-regulated in C1 and C3. In C5, cell communication, Wnt, and metabolism pathways were up-regulated. In C1, cell communication and immune pathways were down-regulated.

### Exploratory Analysis of Cell and Precursor Origins of the Subtypes

These six molecular subtypes were further investigated using GEP data from NT and previously published supervised signatures based on DNA alterations and cellular phenotypes to explore the subtype origins. Based on the growing amount of data suggesting that cancer is closely linked to stem cells, a mouse-derived intestinal stem cell signature [24] and a human colon top and bottom crypt signature were selected and applied to our GEP data [25]. C4 appeared highly enriched for tumors displaying “stem cell phenotype–like” GEPs (91%) and up-regulating of the bottom crypt signature (96%). (Figure S3A). This finding was consistent with the pathways specifically deregulated in C4 (cell cycle pathway down-regulated and cell communication pathway up-regulated).

As previously described for breast cancer [23], we also investigated the existence of a “normal-like” subtype using the GEP centroid from NT samples. C6 was enriched for normal-like GEP tumors, although 86% of them were CIN+.

Serrated CC, in contrast to conventional CC, may arise through a recently introduced serrated neoplasia pathway [27]. We therefore applied the supervised signature, described by Laiho et al. [27], comparing gene expression of serrated to conventional CC to our GEP data. Most of the tumors classified as C2, C3, C4, and C6 displayed a “serrated CC phenotype–like” GEP, whereas those in C1 and C5 displayed a “conventional CC phenotype–like” GEP. A strong association between *BRAF* mutations and the serrated adenoma pathway has been reported [28], and a *BRAF*-mutant-like supervised signature has been described by Popovici et al. [26] that identifies a *BRAF* wild-type subgroup, 30% of which were *KRAS* mutants and 13% of which were double wild-type CC. This signature was also applied to our GEP data: subtypes C2, C3, and C4 were enriched in *BRAF*-mutant-like GEP tumors.

**Figure 3:**
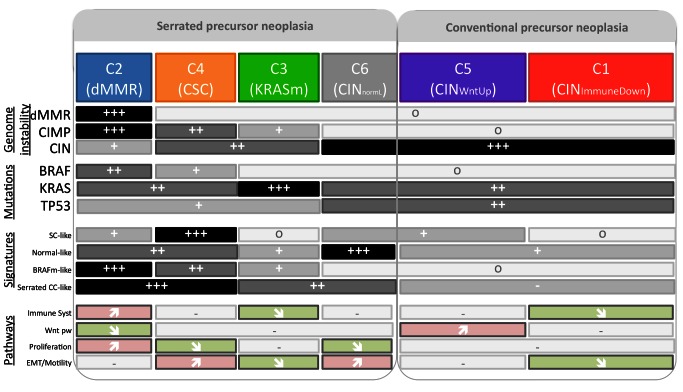
Summary of the main characteristics of the six subtypes. Symbols correspond to the relative frequency within the subtype (o: very low frequencies [∼0%]; +++: very high frequencies; +/++: intermediate frequencies), and arrows indicate significant enrichment of subtype up- and down-regulated genes in most of the pathways of the given category. EMT, epithelial–mesenchymal transition; SC, stem cell; Wnt pw, Wnt pathway.

A schematic summary of the subtype characteristics is shown in Figure 3. The six subtypes were named according their main respective biological characteristic as follows: C1, “CIN_ImmuneDown_”; C2, “dMMR”; C3, “KRASm” (for “*KRAS*-mutant”); C4 “CSC” (for “cancer stem cell”); C5, “CIN_WntUp_”; and C6, “CIN_normL_”.

### Validation of the Subtypes across Nine Colon Cancer Datasets

To validate our six-subtype classification, a 57-gene centroid classifier was built from the discovery set by a 10-fold cross-validation approach (<5% misclassification; Figure S5; Table S5). We applied this signature to the Affymetrix validation set of 1,029 samples (Table 1). All subtypes were found in the same proportions as in the discovery set, and the main associations between the different clusters and anatomoclinical/DNA/GEP characteristics described above were confirmed (Figures S2B and S3B), except for the enrichment of C4 with *BRAF*-mutant and stage IV tumors. When applied to the Agilent TCGA dataset (*n* = 152) [13], the molecular and clinical characteristics of the subtypes were all confirmed (Figure S3C). To further validate the six-subtype classification in the validation dataset, we performed the same consensus clustering approach with the whole validation set; the subtypes generated were highly concordant with the six assigned subtypes (Chi-squared test, *p*<10^−16^).

### Prognostic Value of the Six-Subtype Classification

**Figure 4:**
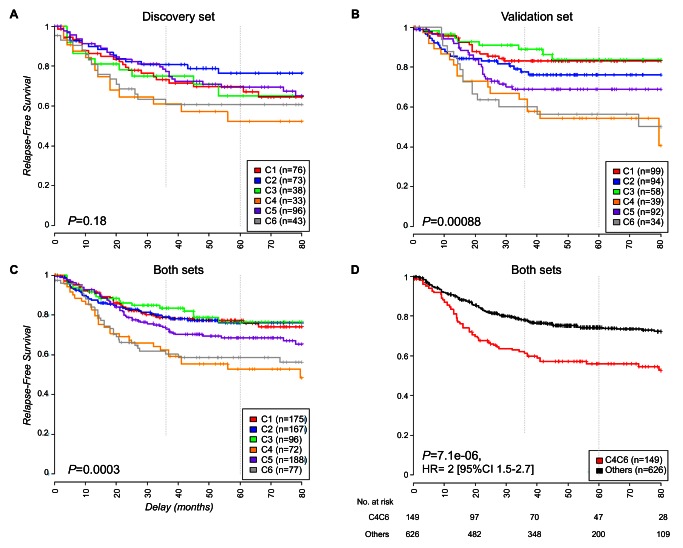
Kaplan-Meier relapse-free survival. This figure shows RFS in (A) the discovery dataset, (B) the validation dataset, (C) the overall dataset, and (D) the overall dataset for C4 and C6 subtypes combined versus the other subtypes; the numbers at risk on the time axis are given.

Further investigation of the clinical relevance of our classification included a prognostic analysis based on RFS restricted to stage II and III tumors. The prognosis of each of our six subtypes in the discovery set (*n* = 359) differed, but not significantly so, with patients whose tumors were classified as C4 and C6 having a relatively poorer outcome (5-y RFS rates of 52% and 61%, respectively, compared to 70%, 77%, 65%, and 70% for C1, C2, C3, and C5, respectively; *p* = 0.18) (Figure 4A). The prognostic value of the six-subtype classification was significant in the validation set (*n* = 416) (*p* = 0.0009), with a worse prognosis confirmed for patients with C4 and C6 tumors (Figure 4B); The six-subtype classification was also significant for the discovery and the validation sets combined (*p* = 0.0003) (Figure 4C). To compare the prognostic value of our classification to other prognostic covariates, we recoded our classification by combining C4 and C6 into a single high-risk group, versus all other subtypes as the low-risk group. This binary classification led to an even stronger association of the high-risk group versus the low-risk group with RFS (hazard ratio [HR] 1.7, 95% CI 1.1–2.6, *p* = 0.014, in the discovery set; HR 2.3, 95% CI 1.5–3.5, *p* = 0.00012, in the validation set; HR 2, 95% CI 1.5–2.7, *p* = 7.1×10^−6^, in the overall dataset) (Figures 4D and S6) and remained an independent prognostic factor, together with TNM stage, in the multivariate analysis (discovery and validation sets analyzed separately and merged) (Tables 2 and S6). The binary classification also remained an independent prognostic factor (*p*<0.01) when common DNA alterations (MMR status, CIMP, and *BRAF* and *KRAS* mutations) were added to the model (Table S7).

**Table 2:** Univariate and multivariate analyses of relapse-free survival according to clinical annotations, the six-subtype classification, and the Oncotype DX prognostic classifier in the overall dataset.

Variables	Value^a^	Univariate Analysis						Multivariate Model 1					Multivariate Model 2				
		*n*	*n* Event	HR	95% CI	Modality *p*-Value (Wald)	Model *p*-Value (Log-Rank)	*n*	HR	95% CI	Modality *p*-Value (Wald)	Model *p*-Value (Log-Rank)	*n*	HR	95% CI	Modality *p*-Value (Wald)	Model *p*-Value (Log-Rank)
TNM stage^b^^,^ c (ref = II)	III	775	202	2	1.5–2.6	0.0000011	0.00000064	775	1.9	1.4–2.5	0.0000077	1.2×10^−09^	775	1.8	1.4–2.4	0.000022	6.0×10^−11^
CIT classification recoded^b^^,^ c (ref = others)	C4C6	775	202	2	1.5–2.7	0.000011	0.0000071		1.8	1.3–2.5	0.00011			1.5	1.1–2.1	0.0097	
CIT classification^b^ (ref = C1)	C2	775	202	1.1	0.66–1.7	0.83	0.0003										
	C3	775	202	0.94	0.54–1.6	0.83											
	C4	775	202	2.3	1.4–3.8	0.00063											
	C5	775	202	1.4	0.89–2.1	0.15											
	C6	775	202	2.1	1.3–3.4	0.0031											
Tumor location (ref = distal)	Proximal	623	173	0.85	0.62–1.1	0.29	0.29										
Sex^b^ (ref = female)	Male	775	202	1.2	0.88–1.5	0.3	0.3										
Age^b^	—	774	202	1	0.99–1	0.84	0.84										
Oncotype DX recurrence score^c^ (ref = low risk)	High risk	775	202	1.9	1.4–2.5	0.0000050	0.0000034							1.6	1.2–2.1	0.0027	

Analyses of RFS were performed using Cox regression. The multivariate models reported correspond to the best multivariate models obtained using a backward–forward selection procedure (R function step). Multivariate model 1 included all given clinical annotations (except tumor location, which was less well filled in) and the classification. Multivariate model 2 included only variables of prognostic interest, i.e., TNM stage, the CIT recoded classification, and the Oncotype DX classifier. Only samples for which all the variables were available were included in multivariate models.

a Value indicates the modality of the annotation associated with the HR.

b Variables used in multivariate model 1.

c Variables used in multivariate model 2.

ref, reference.

### Prognostic Classifiers within Subtypes

The Oncotype DX recurrence score [8] is an emerging prognostic classifier, and we attempted to assess its prognostic value with our data. This score had prognostic value in both the discovery and validation sets, and in the overall dataset (*p* = 3.4×10^−6^; Figure S6). In particular, 97% of the C4 samples were classified as high risk by the Oncotype DX score. However, this score was not prognostic for all of the subtypes (Figure S7). In a multivariate stepwise analysis, both our recoded classification and the Oncotype DX score remained independently prognostic, together with TNM stage (Table 2).

We also attempted an exploratory analysis of the signature described by Salazar et al. [7] by investigating 17 of the 18 probe sets available on the Affymetrix U133 Plus 2.0 chips. We found no significant prognostic value of this 17-gene expression signature in our series (Figure S6).

## Discussion

Using a large comprehensively characterized multicenter cohort of CC patients, we identified six robust molecular subtypes of CC individualized by distinct clinicobiological characteristics. Importantly, this six-subtype classification was validated in nine independent datasets. Furthermore, classification into high- and low-risk subtypes was of prognostic value.

Although retrospective, our cohort was very representative of the clinicopathological characteristics and common DNA alteration frequencies observed in the population of patients with CC.

Our findings clearly demonstrate that anatomoclinical factors and common DNA alterations alone are helpful for highlighting subtype characteristics, but they are not sufficient to define boundaries between subtypes and to describe the molecular heterogeneity of CC. Our classification successfully identified the dMMR tumor subtype, and also individualized five other distinct subtypes among pMMR tumors, including three CIN+ CIMP− subtypes representing slightly more than half of the tumors. As expected, mutation of *BRAF* was associated with the dMMR subtype, but was also frequent in the C4 CIMP+ poor prognosis subtype. *TP53*- and *KRAS*-mutant tumors were found in all the subtypes; nevertheless, the C3 subtype, highly enriched in *KRAS*-mutant CC, was individualized and validated, suggesting a specific role of this mutation in this particular subgroup of CC. There was no significant association between our classification and pathological stage, suggesting that tumor subtype is established at the initial stages.

Exploratory analysis of each subtype GEP with previously published supervised signatures and relevant deregulated signaling pathways improved the biological relevance of the classification. Indeed, this analysis suggested that different types of CC may arise from distinct cell origins, and distinguished between the two main pathways, defined as the serrated and the conventional precursor neoplasia pathways. Interestingly, we not only individualized the dMMR subtype among the serrated precursor neoplasia subtypes, but also within the C4 CSC and the C3 KRASm subtypes. This finding is consistent with the serrated polyp classification showing two main groups: the sessile serrated adenomas, commonly associated with dMMR tumors, and the traditional serrated adenomas, commonly associated with *KRAS*-mutant tumors [29]. However, the proportion of serrated precursor neoplasia tumors that we found was higher than expected, indicating that further pathological investigations are required.

Another interesting finding is the reproducible association between the stem cell signature and the poor prognosis C4 subtype. Almost half of the top genes deregulated in C4—including *secreted frizzled-related protein 2 (SFRP2)*, described as a key factor in stem cell regulation [30] and belonging to the Frizzled gene family, and *growth arrest-specific 1 (GAS1)*—were included in the poor prognosis cluster signature reported by Oh et al. [31]; these genes may therefore be markers of the aggressiveness of CC cells and may constitute potential therapeutic targets.

The C6 CIN_normL_ subtype was more difficult to characterize; it belongs to the CIN+ subgroup but has a GEP and RFS that are distinct from those of the other two CIN subtypes. Several genes up-regulated in C6, in particular *carbonic anhydrase II (CA2)* and *solute carrier family 4, sodium bicarbonate cotransporter, member 4 (SLC4A4)*, were also included in the prognostic classifier described by Lin et al [32].

The two other CIN subtypes, C1 and C5, were more difficult to distinguish from each other. They show common clinical and DNA alteration characteristics. They share some gene expression patterns, leading to lower co-classification rates than for the other subtypes. Moreover, these two subtypes are combined if the number of clusters is set to five instead of six. As a result, the division of C1 and C5 into two distinct subtypes can be questioned (Figure S8). However, the C1 and C5 subtypes are also clearly associated with distinct gene expression signatures (Table S3; Figure S2) and display specific pathway deregulation (immunity and epithelial–mesenchymal transition pathways; Figure 2). In addition, only four out of 507 samples in the validation set classified as subtype C1 or C5 had a mixed assignment C1/C5, as a result of being close to both the C1 and C5 centroids (see Text S1). Altogether, these observations supported these two clusters being representative of two distinct molecular entities.

The biological relevance of our subtypes was highlighted by significant differences in prognosis. In our unsupervised hierarchical clustering, patients whose tumors were classified as C4 or C6 had poorer RFS than the other patients. Thus, our study, like others [7],[31], supports the idea that the unsupervised analysis of transcripts in primary tumors yields information of prognostic value. The prognostic value of our signature was statistically significant in the validation and the overall datasets, independently of TNM stage, with a worse prognosis confirmed for C4 and C6 subtypes. Subtype C4 was enriched in CIMP+ *BRAF*-mutant tumors and may correspond to the poor prognostic cluster reported by Salazar et al. containing the same proportion of *BRAF*-mutant tumors [7].

Prognostic analyses based solely on common DNA alterations can distinguish between risk groups, but are still inadequate, as most CCs are pMMR CIMP− *BRAF*wt (75% in our series; data not shown). Indeed, the markers *BRAF*-mutant, CIMP+, and dMMR may be useful for classifying a small proportion of cases, but are uninformative for a large number of patients. This was illustrated in the study by Salazar et al. in which *BRAF* mutation was found in both good and poor outcome clusters, but was rare in the intermediate prognosis cluster used to build the ColoPrint prognostic classifier [7].

The ColoPrint and Oncotype DX prognostic classifiers were developed recently to improve risk prediction in early-stage CRC [7],[8]. ColoPrint was validated in three independent datasets of stage II–IIIA CC, and the robustness of the signature is currently being evaluated prospectively [33],[34]. The corresponding 17 probe sets available on Affymetrix chips did not identify risk groups in our series (data not shown). Oncotype DX has been validated as a prognostic score in the QUASAR and CALGB9581 trials, and more recently in an independent cohort of patients with stage III CC [35]–[37]. Although not identified by a genome-wide gene expression approach, the Oncotype DX score's prognostic value was confirmed in our overall stage II–III CC dataset but not in every subtype: it had prognostic value for the C3, C4, and C6 subtypes, and marginally in C5; it did not have prognostic value in C1 and C2, which represent 44% of our overall dataset. Our classification added prognostic information that remained significant in the multivariate analysis adjusted for TNM and Oncotype DX score. This suggests that the “one size fits all” prognostic signature approach can be difficult to apply because of the heterogeneity of CC. This may explain, in part, the poor concordance of GEP prognostic signatures in CC [38].

Our multivariate analysis has some limitations. In particular, some established predictors of CC prognosis, notably tumor grade and number of nodes examined, were not included because this information was not available for a substantial proportion of cases [39]. Thus, the significance and robustness of the signature as a prognostic classification requires further confirmation, ideally with large prospective patient cohorts included in adjuvant trials.

In conclusion, we report a new classification of CC into six robust molecular subtypes that arise through distinct biological pathways and represent novel prognostic subgroups. Our study clearly demonstrates that these gene signatures reflect the molecular heterogeneity of CC. This classification therefore provides a basis for the rational design of robust prognostic signatures for stage II–III CC and for identifying specific, potentially targetable markers for the different subtypes.

## Supporting Information

Figure S1 **Discovery and validation sets used in the study.** The data used in this study were collected from the CIT program cohort (a French multicenter cohort) and from publicly available datasets. There were 750 CC samples from the CIT program suitable for common DNA alteration characterization, and 566 of these provided tumor RNA samples satisfying stringent quality control criteria. These RNA were hybridized on an Affymetrix chip (asterisk) and used for molecular subtype determinations. The discovery set was composed of 443 tumors from the CIT cohort. The validation set was composed of the remaining CIT cohort CC samples, CC samples from seven Affymetrix publicly available datasets (indicated with their NCBI GEO accession number), and CC samples from the non-Affymetrix TCGA program (performed on an Agilent platform). For survival analyses, only stage II and III patients were considered, stage I and IV patients not being informative as almost all survive or die, respectively; there were thus 359 cases in the CIT discovery set and 416 in the CIT validation set and three public datasets included in this analysis. pbs, probe sets.
(PDF)

Figure S2 **Heatmaps of subtype-discriminant probe set expression profiles in the discovery set and in the Affymetrix validation set.** (A) Heatmap of the discovery set samples ordered according to gene hierarchical clustering (1 − Pearson metric, Ward linkage) and by subtypes. (B) Heatmap of Affymetrix validation set samples ordered as in (A). For each subtype, discriminant probe sets were selected from the discovery set using a moderated *t*-test, comparing the given subtype to the other subtypes, with an adjusted *p*<10^−5^ and a |log fold change|>0.5, yielding 1,108 discriminant probe sets.
(PDF)

Figure S3 **Associations between molecular subtypes and anatomoclinical characteristics, DNA alterations, and supervised signature annotations in the discovery and validation sets.** Associations were assessed in (A) the Affymetrix discovery set, (B) the Affymetrix validation set, and (C) the TCGA, non-Affymetrix, validation set. For each subtype and variable, the proportion of each modality is represented (dark grey: “positive/true/yes” proportion; white: “negative/false/no” proportion; grey: “data not available” proportion), and the percent of the main feature (dark grey) within each subtype is indicated. The Chi-squared test *p*-values are indicated in red.
(PDF)

Figure S4 **Subtype genomic alteration profiles along the genome.** CC molecular subtypes present different copy-number-change profiles. The profiles were established using genome-wide array-based CGH available for 356 samples. (A) Frequencies of gains (frequency>0) and losses (frequency<0) observed at a given location on the genome are shown for all samples (first row; darker bars are loci with an alteration frequency higher than 20%) and by subtype (darker bars are significantly differentially altered regions, displayed in [B]). (B) Subtype-specific genomic regions of copy-number change. Bars represent significant *p*-values (adjusted *p*-value<0.01), after a logarithmic transformation, for the differences in the proportions of samples with each chromosomal abnormality between the different subtypes. For all samples, regions having an alteration frequency higher than 20% are displayed.
(PDF)

Figure S5 **Determination of subtype prediction centroids.** (A) Percentage of misclassification of the discovery set as a function of the number of top up- and down-regulated gene pairs used in the centroids. Misclassification is computed for the validation set by a 10-fold cross-validation procedure and is plotted by subtype (top) and averaged (bottom). (B) Heatmap of the 57-gene centroids used to assign a new dataset.
(PDF)

Figure S6 **Prognostic value of the recoded CIT classification and of the Oncotype DX–like and Coloprint-like prognostic classifiers in the discovery and validation sets for patients with TNM stage II or III CC.** RFS according to the recoded molecular subtype classification (C4/C6 subtypes versus other subtypes) in each TNM stage category (left, TNM II; middle, TNM III; right, TNM II–III), RFS of high- and low-risk patients as predicted by the Oncotype DX–like classifier, and RFS of patients belonging to cluster 1 and cluster 2 of the ColoPrint 17-gene expression signature in the discovery set (top), the validation set (middle), and the both datasets combined (bottom).
(PDF)

Figure S7 **Prognostic Oncotype DX–like classifier within each CIT molecular subtype in the combined discovery and validation sets.** RFS curves of high- and low-risk patients as predicted by the Oncotype DX–like classifier within each of the six CIT molecular subtypes.
(PDF)

Figure S8 **Selection of the number of clusters.** (A) Cumulative distribution function plot for each tested number of clusters; (B) cumulative distribution function delta area plot; (C) consensus matrix for different numbers of clusters (*k* = 5 to 8).
(PDF)

Table S1 **Patient and tumor characteristics.** CIMP+/−, 3–5 methylated markers/0–2 methylated markers; CIN+/−, CIN>20%/CIN≤20%; F/M, female/male; WT/M, wild-type/mutant.
(XLS)

Table S2 **List of the 1,459 most variant probe sets used to perform unsupervised analysis.** The GeneCluster column corresponds to the gene cluster letters in Figure 1; logFC_CjvsCx corresponds to the gene expression log2 fold changes of subtype Cj versus the other subtypes; adjpv.CjvsCx corresponds to the adjusted *p*-values of the moderated *t*-test comparing Cj versus the other subtypes.
(XLS)

Table S3 **List of the 1,108 subtype-discriminant probe sets.** For each subtype, discriminant probe sets were selected from the discovery set using a moderated *t*-test, comparing a given subtype to the other subtypes (adjusted *p*<10^−5^ and a |log fold change|>0.5), yielding 1,108 discriminant probe sets. The GeneCluster column corresponds to the gene cluster letters in Figure S2; logFC_CjvsCx corresponds to the gene expression log2 fold changes of subtype Cj versus the other subtypes; adjpv.CjvsCx corresponds to the adjusted *p*-values of the moderated *t*-test comparing Cj versus the other subtypes.
(XLS)

Table S4 **Associations of anatomoclinical characteristics, DNA alterations, and supervised signature annotations with the six subtypes based on logistic regression.** Associations were assessed by logistic regression using a multinomial logit model (function mlogit, R package mlogit).
(XLS)

Table S5 **List of the 57 genes used to assign subtypes.** The 57 genes selected to build the subtype predictor, given with each subtype's centroid values.
(XLS)

Table S6 **Univariate and multivariate Cox analyses including the classification and clinical annotations.** Associations of the classification and clinical annotations with RFS were assessed by Cox proportional-hazards regression analyses on (A) the discovery set, (B) the validation set, and (C) both sets. Univariate Cox analyses were performed on each variable independently. The best multivariate model was determined by using a backward–forward selection approach to restrict the multivariate model to the most informative variables for the subset of samples for which all the variables were available. Value indicates the modality of the annotation associated with the HR. H.R., Cox HR.
(XLS)

Table S7 **Univariate and multivariate Cox analyses including the classification and other molecular annotations.** Associations with RFS of the six-subtype classification—including *BRAF*, *KRAS*, and *TP53* mutations, MMR status, and CIMP status—were assessed by Cox proportional-hazards regression analyses on the discovery set. Univariate Cox analyses were performed on each variable independently. The best multivariate model was determined by using a backward–forward approach to restrict the multivariate model to the most informative variables for the subset of samples for which all the variables were available. The *TP53* mutation variable was excluded from the multivariate analysis, as only 201 samples were characterized and as it was not significantly associated to outcome. Value indicates the modality of the annotation associated with the HR. H.R., Cox HR.
(XLS)

Text S1 **Supplementary methods.**
(PDF)

## Abbreviations

CC: colon cancer
CGH: comparative genomic hybridization
CIMP: CpG island methylator phenotype
CIN: chromosomal instability
CIT: Cartes d'Identité des Tumeurs
CRC: colorectal cancer
dMMR: deficient mismatch repair
GEP: gene expression profile
HR: hazard ratio
MMR: mismatch repair
MSI: microsatellite instability
NT: normal tissue
pMMR: proficient mismatch repair
RFS: relapse-free survival
TCGA: The Cancer Genome Atlas
TNM: tumor node metastasis
