# Supplementary figures and images for: Gene Expression Classification of Colon Cancer into Molecular Subtypes: Characterization, Validation, and Prognostic Value
Source: PLoS Med. 2013 May 21;10(5):e1001453. doi: 10.1371/journal.pmed.1001453 (PMC3660251; doi:10.1371/journal.pmed.1001453)

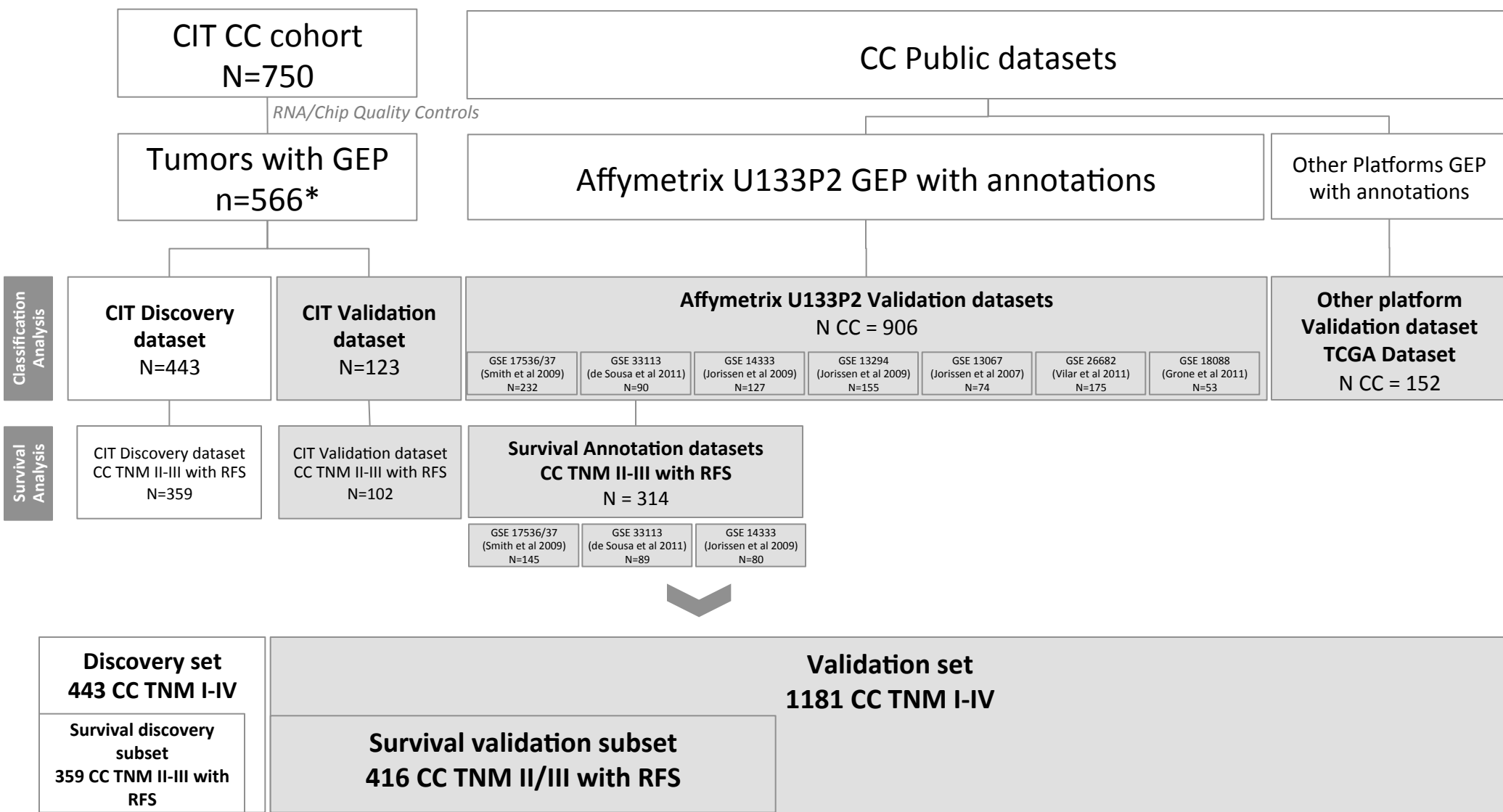

Figure S1

Supplement: Figure S1 — Discovery and validation sets used in the study. The data used in this study were collected from the CIT program cohort (a French multicenter cohort) and from publicly available datasets. There were 750 CC samples from the CIT program suitable for common DNA alteration characterization, and 566 of these provided tumor RNA samples satisfying stringent quality control criteria. These RNA were hybridized on an Affymetrix chip (asterisk) and used for molecular subtype determinations. The discovery set was composed of 443 tumors from the CIT cohort. The validation set was composed of the remaining CIT cohort CC samples, CC samples from seven Affymetrix publicly available datasets (indicated with their NCBI GEO accession number), and CC samples from the non-Affymetrix TCGA program (performed on an Agilent platform). For survival analyses, only stage II and III patients were considered, stage I and IV patients not being informative as almost all survive or die, respectively; there were thus 359 cases in the CIT discovery set and 416 in the CIT validation set and three public datasets included in this analysis. pbs, probe sets. (PDF) [file pmed.1001453.s001.pdf]

A

n=1108  
discriminant  
pbs

Discovery set

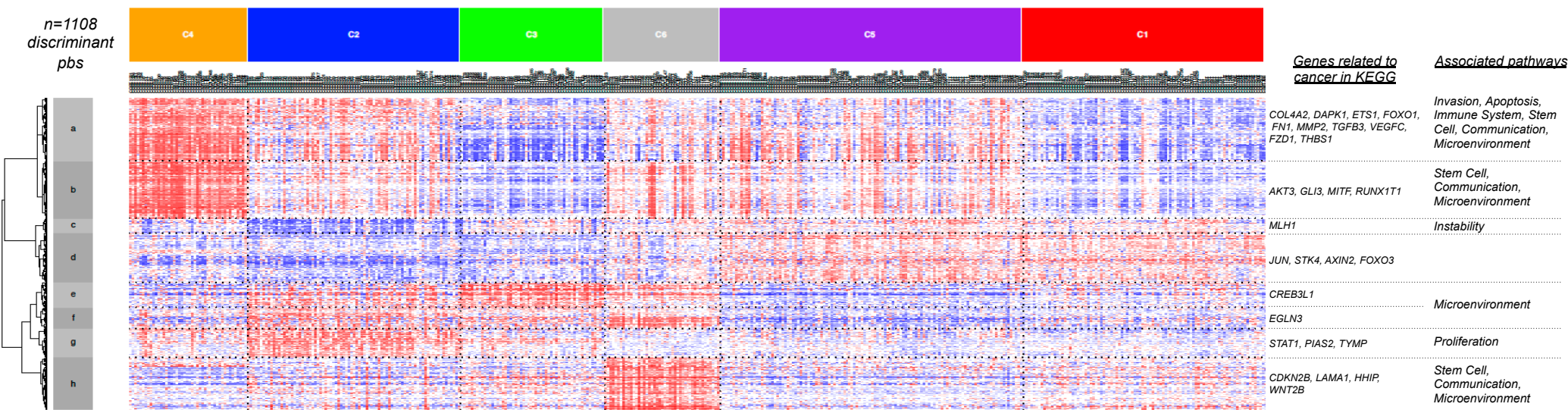

B

n=1108  
discriminant  
pbs

Affymetrix Validation set

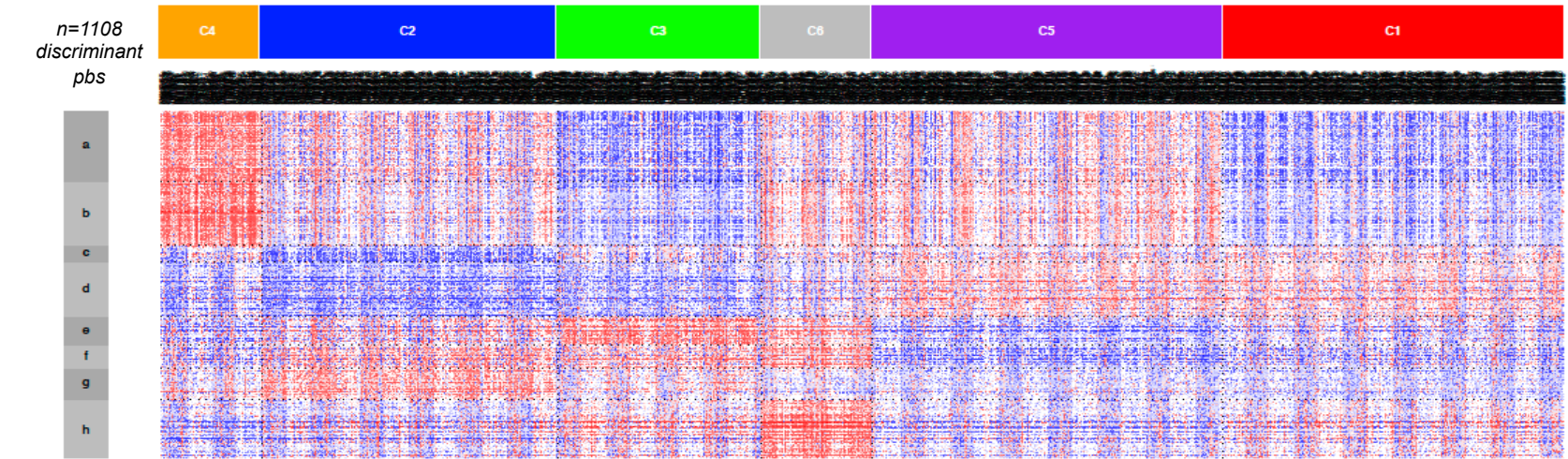

Figure S2

Supplement: Figure S2 — Heatmaps of subtype-discriminant probe set expression profiles in the discovery set and in the Affymetrix validation set. (A) Heatmap of the discovery set samples ordered according to gene hierarchical clustering (1 − Pearson metric, Ward linkage) and by subtypes. (B) Heatmap of Affymetrix validation set samples ordered as in (A). For each subtype, discriminant probe sets were selected from the discovery set using a moderated t-test, comparing the given subtype to the other subtypes, with an adjusted p<10−5 and a |log fold change|>0.5, yielding 1,108 discriminant probe sets. (PDF) [file pmed.1001453.s002.pdf]

A

## Alteration Frequencies

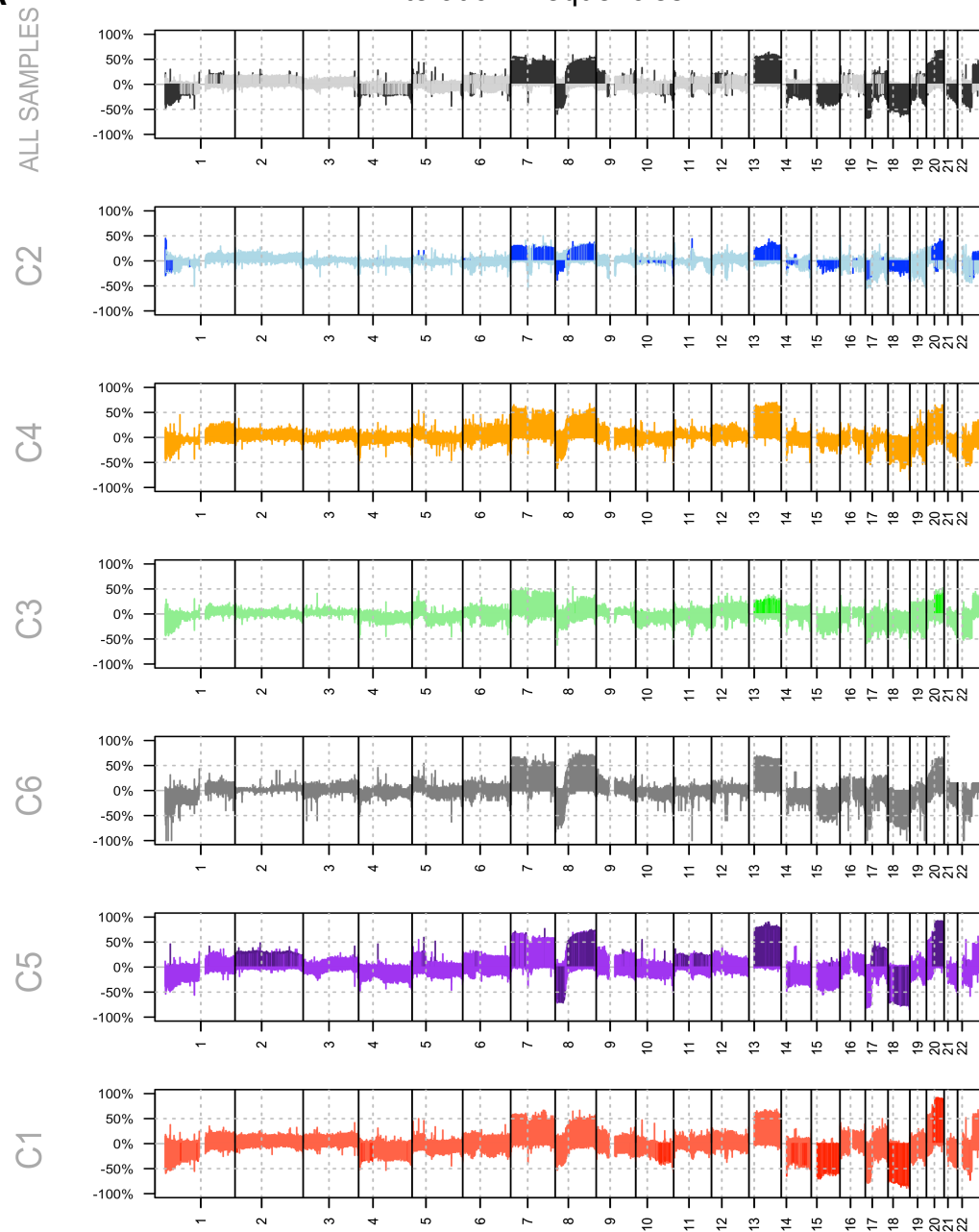

B

## Significant alterations

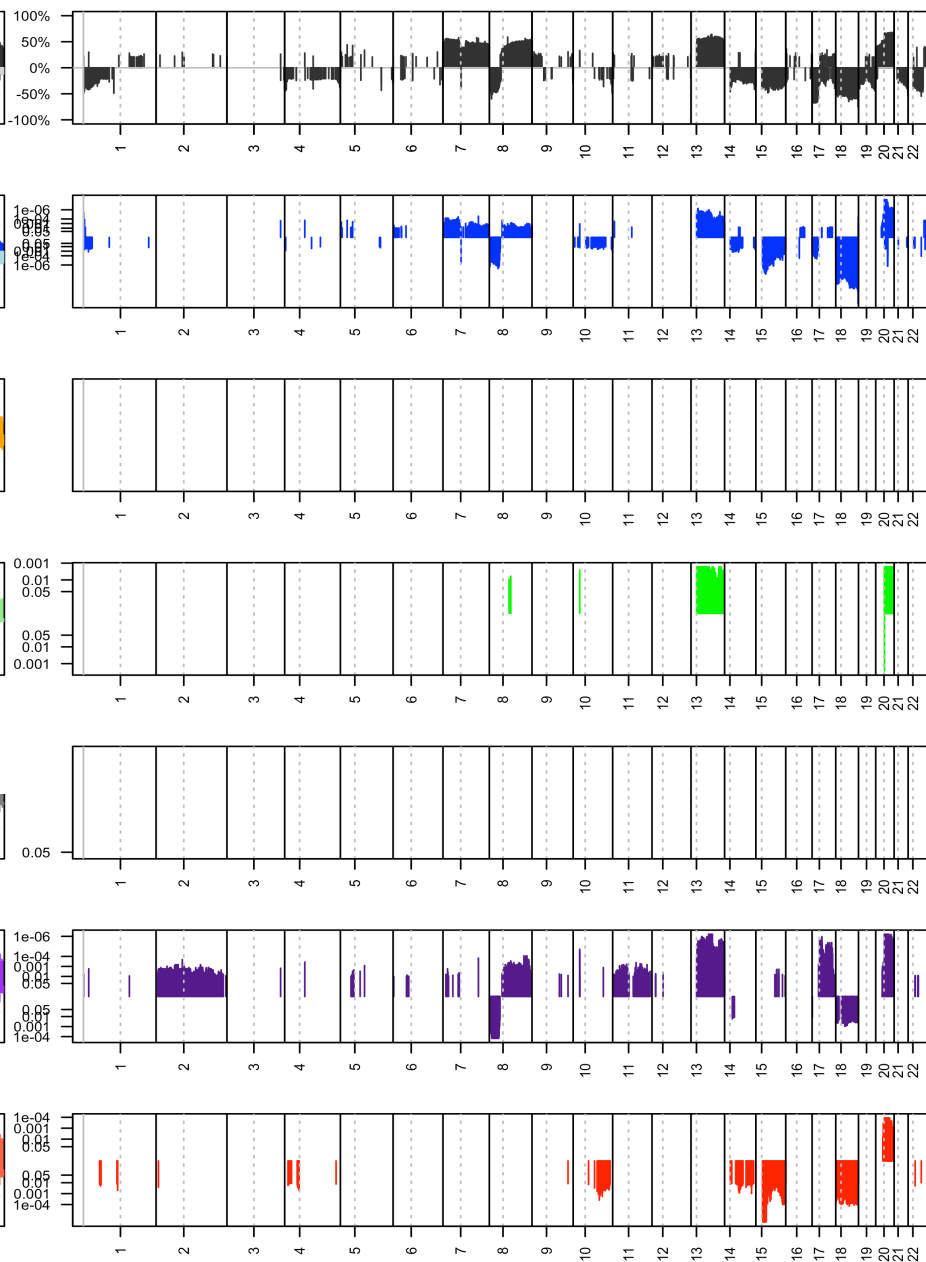

Figure S4

Supplement: Figure S4 — Subtype genomic alteration profiles along the genome. CC molecular subtypes present different copy-number-change profiles. The profiles were established using genome-wide array-based CGH available for 356 samples. (A) Frequencies of gains (frequency>0) and losses (frequency<0) observed at a given location on the genome are shown for all samples (first row; darker bars are loci with an alteration frequency higher than 20%) and by subtype (darker bars are significantly differentially altered regions, displayed in [B]). (B) Subtype-specific genomic regions of copy-number change. Bars represent significant p-values (adjusted p-value<0.01), after a logarithmic transformation, for the differences in the proportions of samples with each chromosomal abnormality between the different subtypes. For all samples, regions having an alteration frequency higher than 20% are displayed. (PDF) [file pmed.1001453.s004.pdf]

A

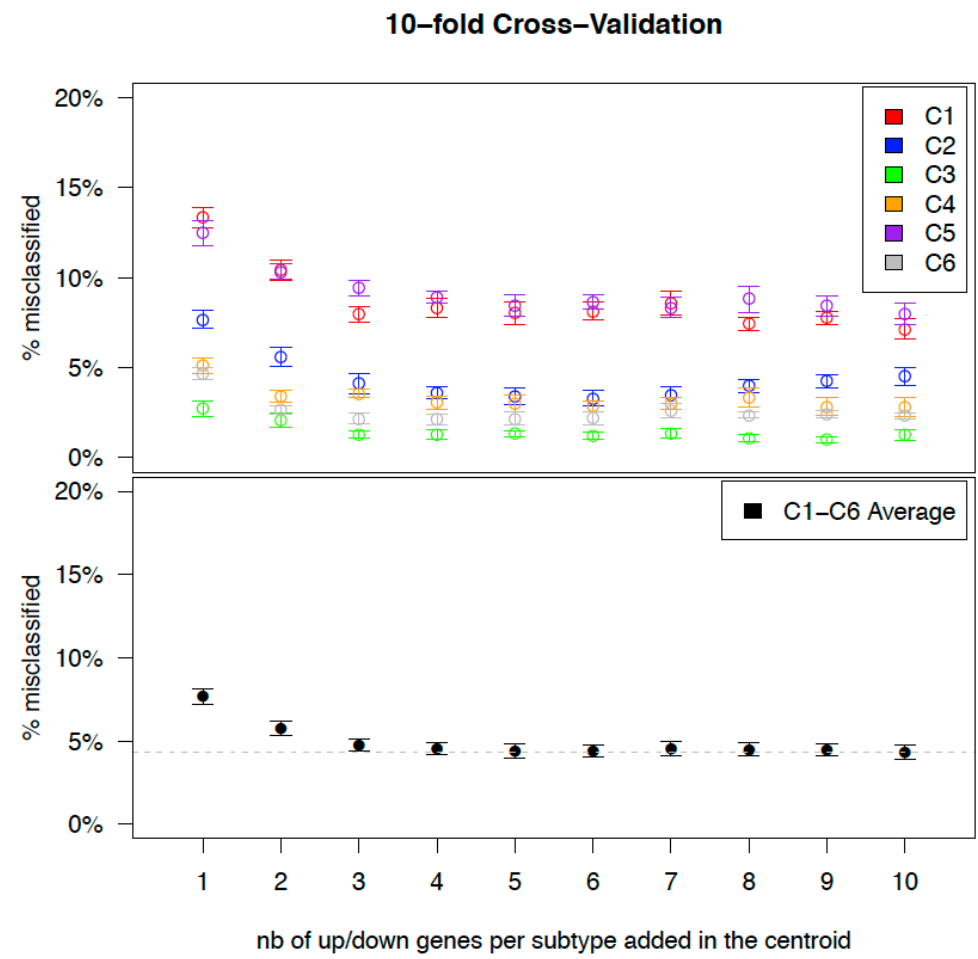

B

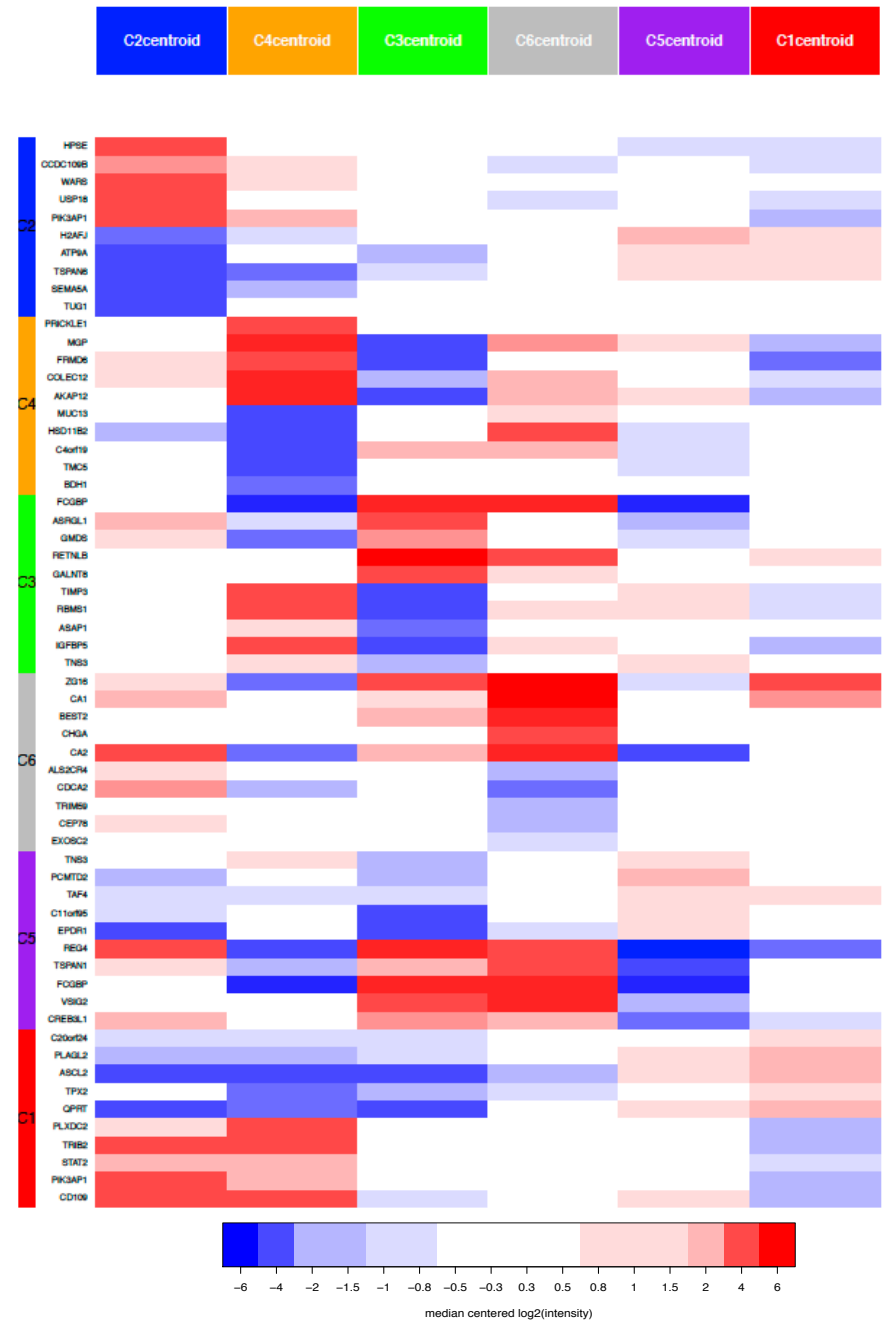

Figure S5

Supplement: Figure S5 — Determination of subtype prediction centroids. (A) Percentage of misclassification of the discovery set as a function of the number of top up- and down-regulated gene pairs used in the centroids. Misclassification is computed for the validation set by a 10-fold cross-validation procedure and is plotted by subtype (top) and averaged (bottom). (B) Heatmap of the 57-gene centroids used to assign a new dataset. (PDF) [file pmed.1001453.s005.pdf]

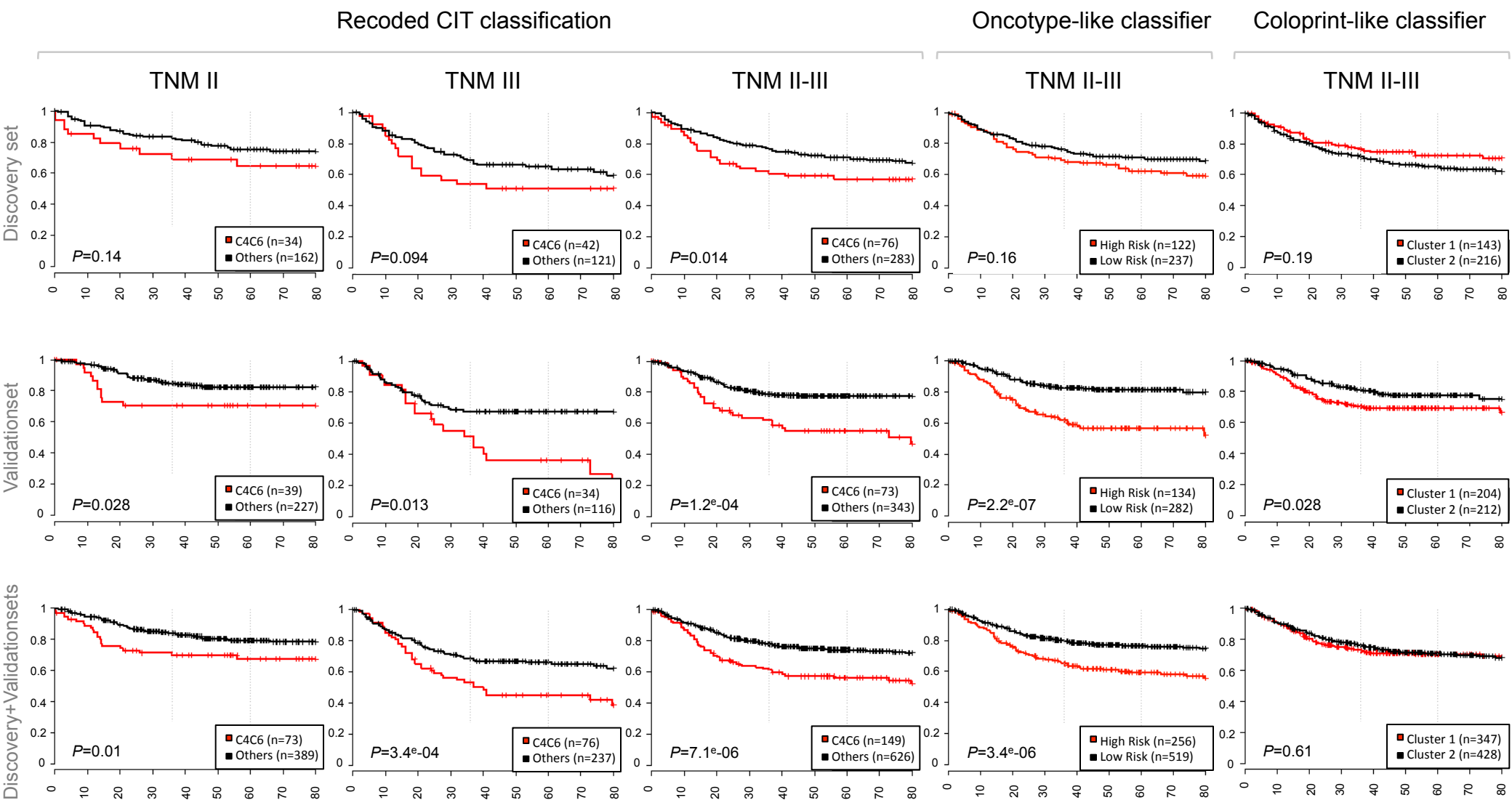

**Figure S6**

Supplement: Figure S6 — Prognostic value of the recoded CIT classification and of the Oncotype DX–like and Coloprint-like prognostic classifiers in the discovery and validation sets for patients with TNM stage II or III CC. RFS according to the recoded molecular subtype classification (C4/C6 subtypes versus other subtypes) in each TNM stage category (left, TNM II; middle, TNM III; right, TNM II–III), RFS of high- and low-risk patients as predicted by the Oncotype DX–like classifier, and RFS of patients belonging to cluster 1 and cluster 2 of the ColoPrint 17-gene expression signature in the discovery set (top), the validation set (middle), and the both datasets combined (bottom). (PDF) [file pmed.1001453.s006.pdf]

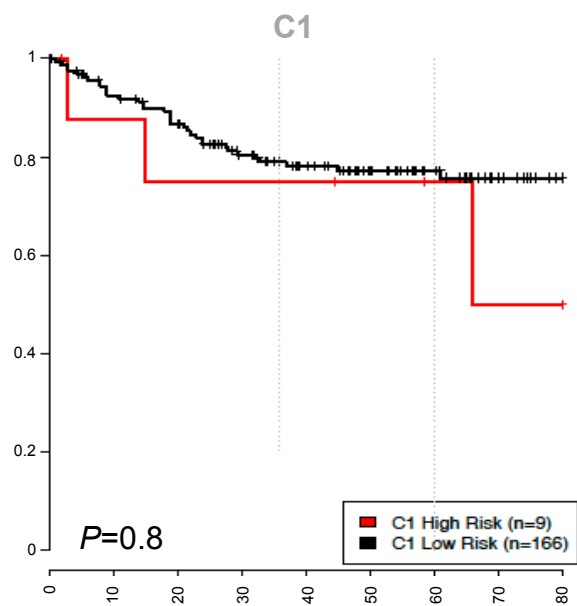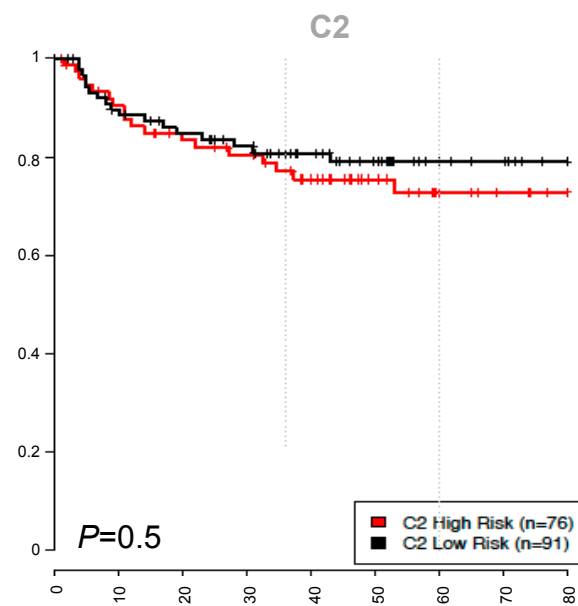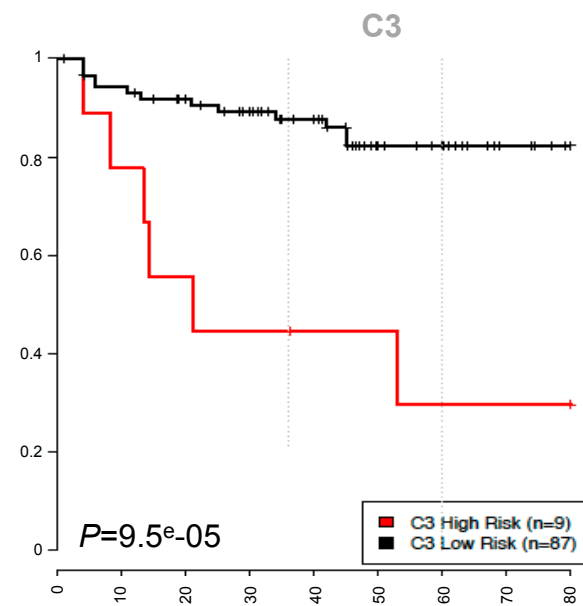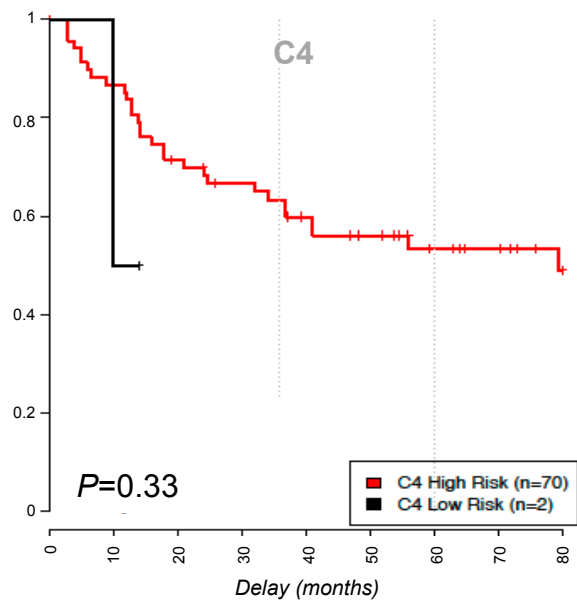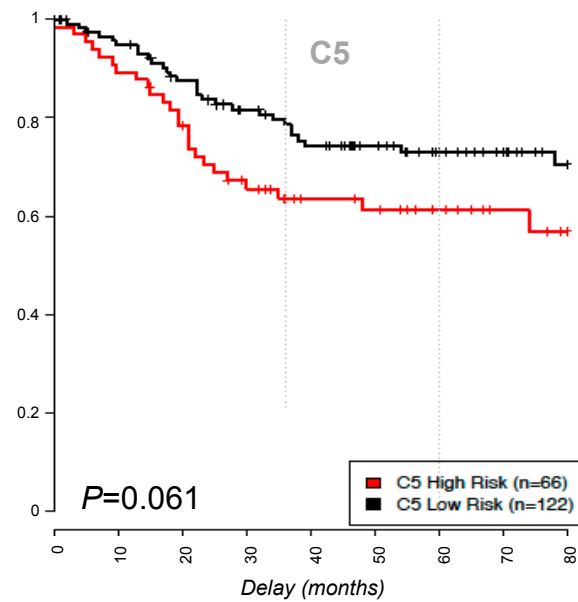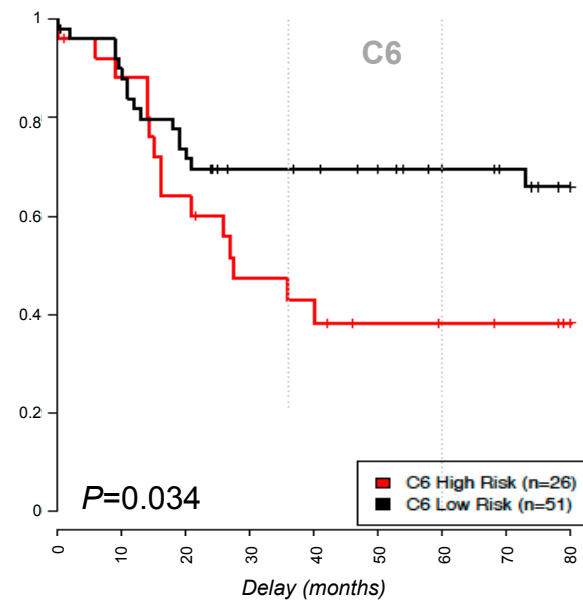

**Figure S7**

Supplement: Figure S7 — Prognostic Oncotype DX–like classifier within each CIT molecular subtype in the combined discovery and validation sets. RFS curves of high- and low-risk patients as predicted by the Oncotype DX–like classifier within each of the six CIT molecular subtypes. (PDF) [file pmed.1001453.s007.pdf]

**A**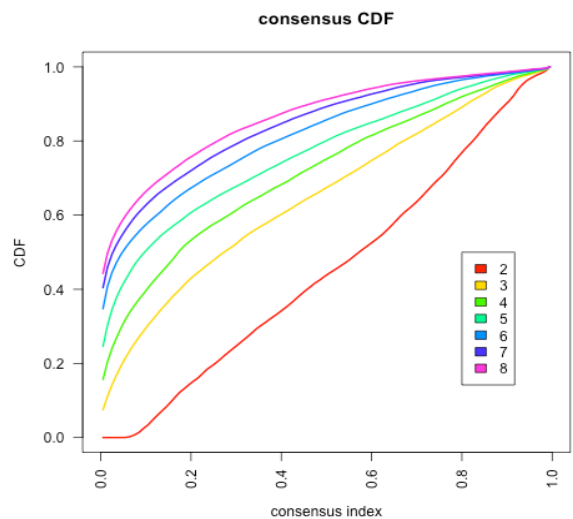**B**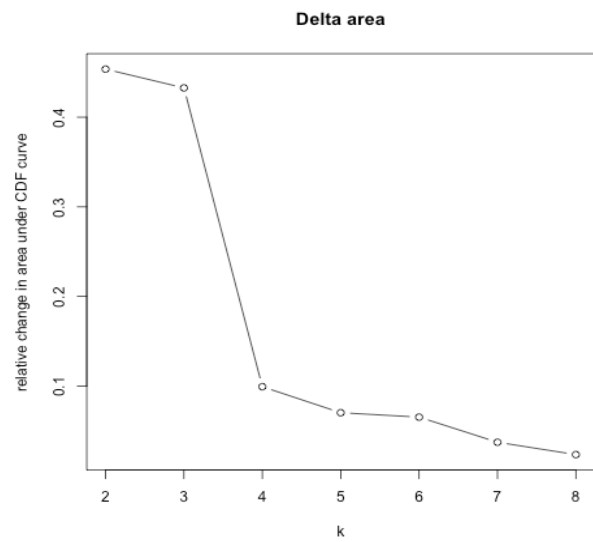**C**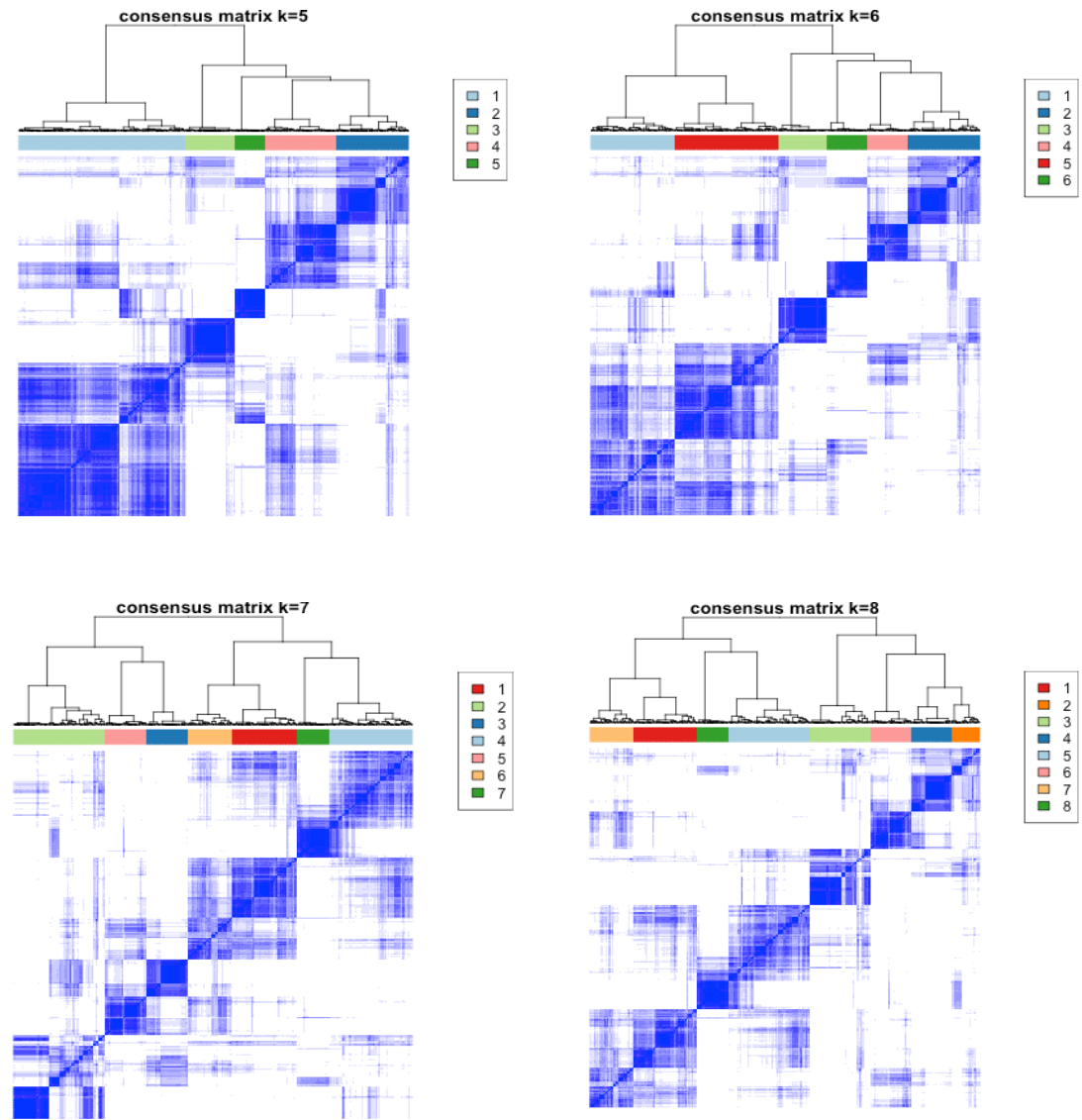**Figure S8**

Supplement: Figure S8 — Selection of the number of clusters. (A) Cumulative distribution function plot for each tested number of clusters; (B) cumulative distribution function delta area plot; (C) consensus matrix for different numbers of clusters (k = 5 to 8). (PDF) [file pmed.1001453.s008.pdf]
