# Supplementary material for: Gene Expression Classification of Colon Cancer into Molecular Subtypes: Characterization, Validation, and Prognostic Value
Source: PLoS Med. 2013 May 21;10(5):e1001453. doi: 10.1371/journal.pmed.1001453 (PMC3660251; doi:10.1371/journal.pmed.1001453)
Supplement: Figure S3 — Associations between molecular subtypes and anatomoclinical characteristics, DNA alterations, and supervised signature annotations in the discovery and validation sets. Associations were assessed in (A) the Affymetrix discovery set, (B) the Affymetrix validation set, and (C) the TCGA, non-Affymetrix, validation set. For each subtype and variable, the proportion of each modality is represented (dark grey: “positive/true/yes” proportion; white: “negative/false/no” proportion; grey: “data not available” proportion), and the percent of the main feature (dark grey) within each subtype is indicated. The Chi-squared test p-values are indicated in red. (PDF) [file pmed.1001453.s003.pdf]

## A Affymetrix Discovery set

| CIT CCMST | | C2 (n=83) | C4 (n=46) | C3 (n=56) | C6 (n=45) | C5 (n=118) | C1 (n=95) |
|---|---|---|---|---|---|---|---|
| dMMR | 2.1e–42 | 68% | 12% | 7% | 0% | 1% | 1% |
| CIMP+ | 1.1e–23 | 59% | 34% | 18% | 3% | 3% | 4% |
| CIN+ | 1.7e–15 | 44% | 73% | 65% | 83% | 95% | 95% |
| BRAF mut | 4.1e–19 | 40% | 22% | 6% | 0% | 1% | 0% |
| KRAS mut | 1.1e–12 | 28% | 50% | 87% | 28% | 27% | 42% |
| TP53 mut | 7.3e–03 | 41% | 45% | 35% | 59% | 71% | 59% |
| Proximal Location | 5e–17 | 72% | 57% | 59% | 16% | 21% | 26% |
| TNM 4 vs 1,2,3 | 5.3e–04 | 5% | 31% | 13% | 2% | 15% | 13% |
| Stem Cell up (Merlos et al) | 2.1e–16 | 42% | 91% | 16% | 47% | 65% | 32% |
| Crypt Bottom up (Kosinski et al) | 1.7e–33 | 27% | 96% | 4% | 67% | 33% | 5% |
| Normal–like GEP | 1.3e–21 | 33% | 30% | 11% | 82% | 17% | 7% |
| BRAFm–like (Popovici et al) | 7.6e–37 | 69% | 50% | 38% | 2% | 3% | 1% |
| Serrated CRC (Laiho et al) | 2.9e–15 | 66% | 74% | 52% | 56% | 24% | 21% |

## B Affymetrix Validation set

| CIT CCMST | | C2 (n=218) | C4 (n=74) | C3 (n=149) | C6 (n=81) | C5 (n=257) | C1 (n=250) |
|---|---|---|---|---|---|---|---|
| dMMR | 1.5e–49 | 80% | 22% | 21% | 16% | 5% | 5% |
| CIMP+ | 6.5e–05 | 48% | 11% | 21% | 0% | 6% | 0% |
| CIN+ | 1.3e–07 | 40% | 67% | 39% | 100% | 97% | 94% |
| BRAF mut | 1.5e–06 | 33% | 0% | 0% | 0% | 0% | 0% |
| KRAS mut | 0.033 | 24% | 46% | 68% | 33% | 25% | 38% |
| TP53 mut | 9.5e–06 | 13% | 60% | 12% | 87% | 66% | 65% |
| Proximal Location | 2.2e–08 | 76% | 46% | 62% | 29% | 33% | 36% |
| TNM 4 vs 1,2,3 | 0.031 | 6% | 13% | 13% | 16% | 19% | 21% |
| Stem Cell up (Merlos et al) | 1.1e–25 | 37% | 88% | 25% | 68% | 64% | 46% |
| Crypt Bottom up (Kosinski et al) | 1.8e–14 | 15% | 50% | 17% | 19% | 14% | 9% |
| Normal–like GEP | 4.6e–39 | 15% | 11% | 20% | 70% | 9% | 9% |
| BRAFm–like (Popovici et al) | 2.6e–95 | 77% | 50% | 46% | 10% | 4% | 3% |
| Serrated CRC (Laiho et al) | 2.1e–50 | 82% | 62% | 54% | 59% | 28% | 18% |

## C TCGA Validation dataset

| CIT CCMST | | C2 (n=36) | C4 (n=7) | C3 (n=20) | C6 (n=6) | C5 (n=38) | C1 (n=45) |
|---|---|---|---|---|---|---|---|
| dMMR | 2.1e–13 | 74% | 17% | 18% | 0% | 0% | 0% |
| CIMP+ | 2.4e–11 | 61% | 29% | 15% | 0% | 5% | 0% |
| CIN+ | 5.8e–03 | 14% | 0% | 0% | 100% | 71% | 78% |
| BRAF mut | 5.8e–10 | 48% | 14% | 5% | 0% | 0% | 0% |
| KRAS mut | 3.8e–03 | 29% | 57% | 74% | 17% | 29% | 27% |
| TP53 mut | 0.017 | 35% | 29% | 26% | 67% | 63% | 62% |
| Proximal Location | 1.2e–05 | 86% | 57% | 65% | 50% | 42% | 27% |
| TNM 4 vs 1,2,3 | 0.33 | 9% | 29% | 10% | 0% | 24% | 16% |
| Stem Cell up (Merlos et al) | 1e–06 | 42% | 86% | 30% | 100% | 76% | 24% |
| Crypt Bottom up (Kosinski et al) | 1.3e–08 | 28% | 71% | 0% | 0% | 3% | 2% |
| Normal–like GEP | 0.023 | 6% | 0% | 0% | 33% | 3% | 4% |
| BRAFm–like (Popovici et al) | 7.3e–05 | 25% | 29% | 5% | 0% | 0% | 0% |
| Serrated CRC (Laiho et al) | 8.6e–09 | 78% | 86% | 70% | 33% | 18% | 22% |

**Figure S3**
