# Supplementary material for: Gene Expression Classification of Colon Cancer into Molecular Subtypes: Characterization, Validation, and Prognostic Value
Source: PLoS Med. 2013 May 21;10(5):e1001453. doi: 10.1371/journal.pmed.1001453 (PMC3660251; doi:10.1371/journal.pmed.1001453)
Supplement: Text S1 — Supplementary methods. (PDF) [file pmed.1001453.s016.pdf]

TEXT S1. SUPPLEMENTARY METHODS

Molecular Characterizations

MMR Status determination

Microsatellite instability was analyzed using a panel of five different microsatellite loci from the Bethesda reference panel [1]. Tumors were characterized on the basis of high-frequency MSI (MSI-H) if two or more of the five markers showed instability, low-frequency MSI (MSI-L) if only one of the five markers showed instability, and MSS if none of the five markers showed instability. MSI-H tumors were further classified as deficient MMR (dMMR), and both MSI-L and MSS as proficient MMR (pMMR).

CIMP Status determination

The CIMP status was determined using the panel of five markers described in Weisenberg *et al* [2]: *CACNA1G*, *IGF2*, *NEUROG1*, *RUNX3* and *SOCS1*. After DNA bisulfite treatment, two multiplex methylation-specific PCR were performed. Fragment analysis was carried out by capillary electrophoresis on automatic sequencer (Beckman Coulter®, Danvers, MA, USA). Methylator phenotype positive cases (CIMP +) had ≥ 3 methylated promoters while CIMP - ones had less than 2 methylated promoters, according to established criteria (22).

Chromosomal Instability (CIN) Status definition

The CIN status was assigned according to CGH alteration profile. A CIN rate was designed as the mean of the per chromosome rate of gained or lost clones (mean(number of clones with a Gain or Loss/total number of clones of the chromosome)). A tumor having an alteration rate superior to 20% was considered CIN+, otherwise CIN-. This cut-off of 20% was chosen based on unsupervised hierarchical clustering of GNL profiles, which delimited a group of tumors with no/very low instability, which displayed a CIN rate inferior to 20%.

Gene expression data normalization

The CIT cohort CEL files were first normalized using the Robust Multi-array Average (RMA) [3] method implemented in the R package affy. Then to remove potential multicenter batch effects, data were corrected using ComBat method [4] implemented in the R package sva, with Centre and RNA extraction method as batch effects and with tumoral and MMR status as features of interest.

Each Affymetrix public datasets used for validation were independently normalized by the RMA method as well.

Molecular subtype determination

Unsupervised Probe set selection

The 1459 probe sets used for subtype determination fulfill the three following criteria:

(1) to be expressed in at least 5% of the samples (i.e. 5th decile of normalized intensities across samples > log2(15))

(2) to have a variance significantly different from the median variance of all probe sets (i.e. variance test *p*-value<0·01)

> Variance test: For each probe set (P) we tested whether its variance across samples was different from the median of the variances of selected probe sets in (1). The statistic used was ((n-1)×Var(P) / Var_{med}), where n refers to the number of samples. This statistic was compared to a percentile of the Chi-squared distribution with (n-1) degrees

of freedom (this criteria is used in the BRB ArrayTools filtering tool, described in the User's Manual [5]) and yielded a *p*-value for each probe set.

(3) to have a high robust coefficient of variation (rCV > 0·186).

rCV : rCV for each probe set was calculated by dividing the standard deviation by the mean, eliminating the highest and lowest expression value across the samples for each probe set.

rCV threshold determination : the cut-off point was defined using Gaussian mixture model clustering approach (R package mclust [6]) which defined 4 groups of rCV, the most variant one containing 1459 probe sets, the minima rCV being 0·186.

Consensus Unsupervised class discovery approach

The subtypes were determined using the consensus clustering approach described in Monti et al [7] and implemented in the R package ConsensusClusterPlus [8]. In brief, a clustering analysis is performed n times on subsets of the probe sets and of the samples selected randomly. Then all derived partitions for a given number of clusters k are summarized by clustering the (samples x samples) co-classification matrix*. The whole data were first gene median centered and the parameters used were set as follows:

- Clustering algorithm: hierarchical clustering
- Clustering metrics: (1-Pearson correlation) distance and Ward linkage
- n resamplings: 1000
- Proportion of samples and probe sets used in each resampling: 90%,
- k tested: from 2 to 8.

As described in Monti et al, [7] the choice of the number of clusters can be based on the delta area plot and should correspond to the number of clusters k where the Cumulative distribution (CDF) levels off and the corresponding relative increase in the CDF area gets closes to zero. Following this procedure, in our case, several values of k could reasonably have been selected (Figure S7), and, at inspecting the consensus matrices progression as suggested, the more balanced partition appeared to be for k=6.

* giving for each pair of samples the proportion of partitions in which these two samples were co-clustered.

Molecular subtype prediction

To assign a subtype to each sample from the validation series, we developed a centroid-based predictor using the most discriminating probe sets (over and under expressed) of each subtype.

The selection of the probe sets used in the centroids was performed among the probe sets selected in the 2 first steps of the subtype determination approach and having an Affymetrix grade A annotation (NetAffx [9] Annotations version na31 were used) and then as follows for each subtype:

- Probe sets significantly differentially expressed in samples of the given subtype compared to samples of other subtypes according to the Limma moderated t-test [10] or the Welch t-test (adjusted *p*-value<1e-5 and |log2 fold change|>0·5) were retained
- Then the selected probe sets were ordered according to their AUC score (computed using the R package PresenceAbsence [11]) and only those with a score superior to 0.7 were kept.
- To avoid the selection of highly correlated probe sets (redundancy) we clustered probe sets using hierarchical clustering (distance=1-Pearson, linkage method=Ward), cut the

tree to isolate uncorrelated clusters (tree cut-off (1-correlation) = 0.9) and kept one probe set per cluster, the one having the best AUC and a gene symbol annotation.

- To select the probe sets to use in the centroid, we proceeded by a 10-fold cross-validation approach. The discovery dataset was split into 10 subsets. The top up/down regulated pairs of probe sets were used to build centroids on 9 of the 10 subsets and the assignment (see below) was then computed on the remaining subset. This procedure was repeated for each subset and for each number of probe set pairs tested (from 1 to 10). The lowest global misclassification was obtained for 5 top up/down pairs (Figure S4A).

This procedure yields 57 probe sets (corresponding to 57 unique genes), 3 probe sets being specific to several subtypes but with inverted regulation (Table S2, Figure S4B).

Then using those probe sets, 6 centroids were computed on the gene-median centered discovery dataset and for each validation dataset (RMA normalized and gene-median centered), the distance to the 6 centroids of each sample was computed and samples were assigned to the closest centroid subtype. The decision rule was based on the diagonal quadratic discriminant analysis method (DQDA) and is defined as follows:

$$DQDA(X) = \underset{j\in\{C1,\dots,C6\}}{Arg\,min}\left(\sum_{i=1}^{N}\frac{\left(x_i-\mu_{j,i}\right)^2}{v_{j,i}}\right)+\sum_{i=1}^{N}\log(v_{j,i})$$

where N is the number of genes (here N=57), x the expression normalized values, $\mu_{j,i}$ and $\nu_{j,i}$ the mean and the variance of the gene i across samples of the subtype j from the discovery data set (i.e. the centroid).

The confidence of the prediction was evaluated by identifying outliers (too distant samples) and mixed assignment samples (when a sample is close to several centroids). More specifically, a sample is said to be an outlier if its distance to the closest centroid is superior to n times the median absolute deviation (mad) of the distances of the samples used to compute the centroid; n is defined as the maximum (distances to centroid-median$_{\text{distances to centroid}}$)/mad$_{\text{distances to centroid}}$). A sample has a mixed assignment if the difference of its distance to centroid is inferior to the 1st decile of the difference between centroids on data used to compute centroids.

Among the 1029 samples of the validation data set, only 13 samples had an uncertain assignment and no outliers were found.

The subtype prediction procedure is implemented in the R package citccmst that will be available at the R CRAN repository (http://cran.r-project.org/).

N.B.: This prediction procedure has been designed from Affymetrix U133P2 data set and applied to Affymetrix U133P2 data sets so the prediction of other platform datasets should require caution and adjustment (as gene symbol mapping, re-computing the centroid using those selected genes and using another distance metrics).

Molecular subtype characterization

i) Non-tumoral Colonic Mucosa GEP tumors:

To evaluate the similarity of GEP tumors to colon normal tissue, the distance of each tumor samples to the centroid of the 1459 probe sets of the normal mucosa samples was computed.

A tumor was assigned Normal-like GEP if its distance was amongst the 25% closest to the NC centroid (metrics 1-Pearson correlation, Ward linkage, median gene centered data).

ii) Annotation with published supervised signatures

Tumors were assigned to molecular and cellular phenotypes as follows:
For all signatures used, genes were matched to our probe sets by the Gene Symbol annotation and only the most variant probe set (maximal rCV) was selected.

Stem cell signature up regulated tumors:
The signature used is the Merlos-Suarez et al [12] Intestinal Stem Cells (ISC) signature (in their table S1). As describe in their article, an ISC score was computed by gene centering the data (median) and computing the mean expression of all genes of the signature. A tumor was assigned Stem Cell signature up regulated when this score was superior to the mean of all scores.

Cell from crypt signature up regulated tumors:
The signature used is composed of a selection of the genes highly up regulated in bottom crypt given in Kosinski et al [13] (in their table 3, *p*-value paired t-test < 1e-5 and |logFC|>2). As only some of those genes were highly up regulated in our tumors, a hierarchical clustering approach was preferred over mean expression score and allows us to divide our samples into 2 groups, those with a subset of those bottom gene highly up regulated were assigned Crypt Cell Signature up regulated.

Popovici BRAF mutated like tumors:
As described in their article [14], the genes given in the Table 2 were used and if the mean of G1 genes was smaller than the mean of G2 the tumor was assigned *BRAF*m-like.

Laiho et al Serrated CRC tumors:
A centroid of the probe sets of their signature [15] (Table S3) was computed on the original data set (GSE4045) and our tumors were assigned Serrated or Conventional adenoma depending on the distance to the closest centroid (metrics 1-Pearson correlation, median gene centered).

iii) Cancer pathway analysis

KEGG pathways and some gene sets from Gene Ontology selected to be related to cancer hallmarks (Cell communication, growth/death, Immune system, Motility, Replication and repair, Angiogenesis, Metabolism and main cancer signal transduction pathways) were tested for enrichment of the top 1000 up and top 1000 down regulated genes of every subtypes (genes were selected based on Limma t-test *p*-values and |FC|>1·5) by computing a hypergeometric test (*p*-value <0·05).

iv) CGH alteration frequency profiles

CGH array chip and experiment have already been described here [16]. Raw log2-ratio values were filtered (i) using a signal-to-noise threshold of 2·0 for the reference channel and (ii) when the individual single intensities for the sample or reference was less than 1·0 or at saturation (i.e. 65,000). The remaining values were normalized using the lowess within-print tip group method [17] and the values of clone replicates were averaged if their standard deviation was less than 0.25 otherwise filtered. Then to define region of loss and gain, for each sample the normalized values were smoothed to obtain segments using tilingArray method [18] and the DNA copy number was determined as follows**:** the level (L_N)

corresponding to a normal (i.e. diploid) copy number is determined as the first mode of the distribution of the smoothed log2-ratio values across all autosomes; the standard deviation (SD) of the difference between normalized and smoothed values is calculated; then for all clones in a segment, the 'GNL' copy number status (G: gain - N: normal - L: loss) is determined based on the segment smoothed log-ratio value (X): if $X > L_N + SD$ then status=gain (G), if $X < L_N - SD$ then status=loss (L), else status=normal; in a given segment, outlier clones that yielded normalized log2-ratio values (Y) such that $Y > L_N + 3 \times SD$ (respectively $Y < L_N - 3 \times SD$) are classified as gains (respectively losses).
Alteration frequencies profiles in Figure S3 were obtained using the 356 CGH arrays available for samples of the discovery dataset and by computing the proportion of samples harbouring a gain or a loss of copy, at each clone of the array for all samples and by subtypes. Frequently altered genomic regions (Figure S3 B) in the whole dataset were determined by identifying regions for which the proportion of alteration (in gain or loss) exceed 20%. Subtypes specific regions were determined by applying at each clone a test of proportion comparing the proportion of alteration (gain and loss) in the samples of a given subgroup versus in samples of the others corrected for multiple-testing by FDR (Benjamini and Hochberg) [19], a subtype specific genomic regions being defined as a set of consecutive clones significantly more altered in the subtype of interest (*p*-values < 0·01).

Molecular Subtype Robustness

Internal robustness:

- The subtypes were obtained using a consensus clustering procedure using both gene and sample resampling (1000 random subselections of 90% of the samples and 90% of the genes), such that these results are stable under conditions of gene and sample resampling.
- The subtypes were obtained from a large set (n=443) of samples processed with the same experimental procedure, as part of the Cartes d'Identité des Tumeurs program.
- Moreover, we have tested that our classification results were also repeatedly obtained using different metrics (Euclidian/Pearson).

External robustness:

The subtypes were validated on a large dataset collected under different conditions, from numerous centers: clinical and biological characteristics of the subtypes were found to be conserved in this validation set.

Survival Analyses

Survival analyses were restricted to the subgroup of patients with stage II-III tumors. Additional prognostic biomarkers are most needed for these patients. This is because the vast majority of stage I CRC patients will never relapse after curative surgery and will not derive benefit from adjuvant chemotherapy because the prognosis is excellent. Also, most stage IV CRC patients are already metastatic and will die from their disease.

Relapse-Free Survival was used and defined as the time from surgery to the first recurrence.

Survival curves were obtained according to the method of Kaplan and Meier (function Surv, R package survival) and differences between survival distributions were assessed by Log-rank test using an endpoint of five years/60 months (function survdiff, R package survival). The proportional-hazards assumption was tested to examine the model's appropriateness (function cox.zph, R package survival).

For the analysis of associations with patient outcome, univariate and multivariate models were computed using Cox proportional-hazards regression (function coxph, R package survival). Univariate analyses were performed to assess the marginal value of each variable independently from the others. For multivariate analyses, first a multivariate analysis using all variables (excluding those with insufficient data as not to reduce the power of the analysis) was performed. Next, to select the best multivariate model, a backward-forward step procedure was computed to restrict the multivariate model to the most informative variables as described in Venables & Ripley, 2002[20] (function *step(), R package stats*). Only samples for which all the variables were available were included in multivariate models.

Recurrence Risk group assignment according to O'Connell and Salazar predictors

O'Connell et al [21] Oncotype classifier:

The O'Connell Recurrence Risk (RS) score is composed of 12 genes among which 5 reference genes and 7 genes associated to recurrence. For the reference genes, when several probe set were possible, the less variant one was selected. For the other genes, data were median gene centered and aggregated by mean if several probe sets were available. Then the recurrence genes intensities for each sample were subtracted by the mean of the reference gene per sample and the formula given in O'Connell et al (Figure 3 and supplemental method) was applied for each sample RS_u = 0·15* mean(BGN,FAP,INHBA)- 0·3*mean(MKI67,MYC,MYBL2)+ 0·15* GADD45B)

This score was then rescaled RS=44*(RSu+0.82). RS ranged from 8 to 82 so ranging its distribution between 0 and 100 was not necessary. A tumor was predicted with high risk if the score was superior or equal to 41 as mentioned in the article.

Salazar et al [22] predictor:

Among the 18 genes from their classifier, only 17 are found in Affymetrix annotations. As no centroid was available and down/up regulations were not mentioned, we computed a hierarchical clustering of the probe sets average matching those 17 genes to obtain 2 clusters.

References

1. Boland CR, Thibodeau SN, Hamilton SR, Sidransky D, Eshleman JR, et al. (1998) A National Cancer Institute Workshop on Microsatellite Instability for cancer detection and familial predisposition: development of international criteria for the determination of microsatellite instability in colorectal cancer. Cancer Res 58:5248-57.

2. Weisenberger DJ, Siegmund KD, Campan M, Young J, Long TI, et al. (2006) CpG island methylator phenotype underlies sporadic microsatellite instability and is tightly associated with *BRAF* mutation in colorectal cancer. Nat Genet 38:787-93.

3. Irizarry RA, Hobbs B, Collin F, et al. (2003) Exploration, normalization, and summaries of high density oligonucleotide array probe level data. *Biostatistics* 4:249-264.

4. Johnson WE, Li C, Rabinovic A (2007) Adjusting batch effects in microarray expression data using empirical Bayes methods. Biostatistics 8: 118–127.

5. Simon R, and Peng Lam A. (2003) BRB-ArrayTools software v3.1 User's Manual linus.nci.nih.gov/BRB-ArrayTools.html.

6. Chris Fraley and Adrian E. Raftery (2006) MCLUST Version 3 for R: Normal Mixture Modeling and Model-based Clustering.Technical Report No. 504, Department of Statistics, University of Washington (revised 2009)

7. Monti S, Tamayo P, Mesirov J, Golub T (2003) Consensus clustering: A resampling-based method for class discovery and visualization of gene expression microarray data. *Machine learning* 52:91-118.

8. Matt Wilkerson (2011). ConsensusClusterPlus: ConsensusClusterPlus. R package version 1.6.0.

9. Liu G, Loraine AE, Shigeta R, Cline M, Cheng J, Valmeekam V, Sun S, Kulp D, Siani-Rose MA (2003) NetAffx: Affymetrix probesets and annotations. *Nucleic Acids Res*;31(1):82-6.

10. Smyth, G.K. (2004) Linear models and empirical bayes methods for assessing differential expression in microarray experiments. *Stat Appl Genet Mol Biol* 3, Article3

11. Freeman, Elizabeth (2007) PresenceAbsence: An R Package for Presence-Absence Model Evaluation. USDA Forest Service, Rocky Mountain Research Station, 507 25th street, Ogden, UT, USA.

12. Merlos-Suárez A, Barriga FM, Jung P, et al. (2011) The intestinal stem cell signature identifies colorectal cancer stem cells and predicts disease relapse. *Cell Stem Cell* 8:511-24.

13. Kosinski C, Li VS, Chan AS, Zhang J, et al. (2007) Gene expression patterns of human colon tops and basal crypts and BMP antagonists as intestinal stem cell niche factors. *Proc Natl Acad Sci U S A* 104:15418-23.

14. Popovici V, Budinska E, Tejpar S, et al. (2012) Identification of a poor-prognosis *BRAF*-mutant-like population of patients with colon cancer. *J Clin Oncol* 30:1288-95.

15. Laiho P, Kokko A, Vanharanta S, et al. (2007) Serrated carcinomas form a subclass of colorectal cancer with distinct molecular basis. *Oncogene* 26:312-20.

16. Guedj M, Marisa L, de Reynies A, et al. (2012) A refined molecular taxonomy of breast cancer. *Oncogene* 1;31(9):1196-206.

17. Yang YH, Dudoit S, Luu P, et al. (2002) Normalization for cDNA microarray data: a robust composite method addressing single and multiple slide systematic variation. *Nucleic Acids Res* 30:e15.

18. Wolfgang Huber and Joern Toedling and Lars M. Steinmetz (2006) Transcript mapping with high-density oligonucleotide tiling arrays. *Bioinformatics* 22, 1963-1970.

19. Benjamini Y and Hochberg Y (1995). Controlling the false discovery rate: a practical and powerful approach to multiple testing. *J. Roy. Statist. Soc. Ser. B*. 1995; 57 289-300.

20. Venables, W. N. and Ripley, B. D. (2002) _Modern Applied Statistics with S. Fourth edition. Springer.

21. O'Connell M, Lee M, Lopatin M, et al. (2012) Validation of the 12-gene colon cancer recurrence score (RS) in NSABP C07 as a predictor of recurrence in stage II and III colon cancer patients treated with 5FU/LV (FU) and 5FU/LV+oxaliplatin (FU+Ox). *J Clin Oncol* 30 (suppl; abstr 3512).

22. Salazar R, Josep Tabernero, Victor Moreno, et al. (2012) Validation of a genomic classifier (ColoPrint) for predicting outcome in the T3-MSS subgroup of stage II colon cancer patients. *J Clin Oncol* 30 (suppl; abstr 3510).
